# Association Between Non-alcoholic Fatty Liver Disease and Risk of Stroke: A Systematic Review and Meta-Analysis

**DOI:** 10.3389/fcvm.2022.812030

**Published:** 2022-03-08

**Authors:** Meng Wang, Ben-Gang Zhou, Yi Zhang, Xi-Fang Ren, Ling Li, Bo Li, Yao-Wei Ai

**Affiliations:** ^1^Department of Neurology, The Third Clinical Medical College of China, Three Gorges University, Gezhouba Central Hospital of Sinopharm, Yichang, China; ^2^Department of Gastroenterology, The First People's Hospital of Yichang, The People's Hospital of China Three Gorges University, Yichang, China; ^3^Beijing Hospital of Traditional Chinese Medicine, Capital Medical University, Beijing Institute of Chinese Medicine, Beijing, China

**Keywords:** non-alcoholic fatty liver disease, stroke, meta-analysis, systematic review, non-alcoholic steatohepatitis

## Abstract

**Background/Objectives:**

Recent observational studies have explored the association between non-alcoholic fatty liver disease (NAFLD) and stroke with controversial results. We therefore performed a meta-analysis to investigate this possible association.

**Methods:**

PubMed, EMBASE and Web of Science database were searched from inception until December 2019, and updated on May 2021. Random-effects meta-analyses were performed by generic inverse variance method. Subgroup and sensitivity analyses were also conducted. The PROSPERO registered number of this study is CRD42020167330.

**Results:**

Twenty observational (15 cohort, 4 cross-sectional, and 1 case-control) studies with 17,060,388 participants were included in the meta-analysis. Meta-analysis of data from 18 studies with 17,031,672 participants has shown that NAFLD was associated with mildly increased risk of stroke (OR = 1.18, 95% CI: 1.08–1.30, *P* = 0.0005). Similar results were observed in most of the subgroup analyses we performed. Sensitivity analyses did not alter these findings. Meta-analysis of data from 3 studies with 29,614 participants has shown that insufficient evidence to support the proposed association between NAFLD-fibrosis and an increased risk of stroke.

**Conclusions:**

We found that NAFLD was associated with increased risk of stroke. However, there was insufficient evidence to support the proposed association between NAFLD-fibrosis and an increased risk of stroke. To better understand any association, future well-designed prospective studies that take fully account of specific population, type of stroke, and confounding factors are warranted.

**Systematic Review Registration:**

Unique Identifier: CRD42020167330.

## Introduction

Non-alcoholic fatty liver disease (NAFLD) is the most prevalent chronic liver disorder globally, with high prevalence of about 25% worldwide, 29.6% in Asia, 30.5% in South America, and 31.8% in the Middle East (1, 2). NAFLD encompasses a spectrum of histopathological features, ranging from simple non-alcoholic fatty liver (NAFL) to non-alcoholic steatohepatitis (NASH), progressing to liver fibrosis, cirrhosis, and ultimately to hepatocellular carcinoma (3, 4). In view of the increasing global epidemic of obesity and type 2 diabetes (T2DM), which were closely related to NAFLD, recent research models predict that the prevalence of NAFLD will continue to increase, and subsequently lead to tremendous clinical and high economic burden (5, 6). Nevertheless, the burden of NAFLD is not only limited to progressive liver disease, but also associated with an increased risk of extrahepatic complications (such as cardiovascular disease, colorectal tumors, and chronic kidney disease) (7–9).

In this context, the association between NAFLD and stroke has recently attracted considerable attention. Stroke, a concerning disease globally, is the second largest cause of death in the world and the second most common cause of global disability-adjusted life-years (DALYs, 116.4 million) (10). Although age-standardized mortality rates from stroke have decreased dramatically from 1990 to 2016, the overall burden of stroke remains high and continues to increase due to the growing and aging population, and is unlikely to reduce without interventions to deal with stroke risk factors (10, 11). Herein, it is essential to explore novel and potentially modifiable risk factors for stroke.

In recent years, a large number of observational studies (12–31) have explored the relationship between NAFLD and stroke, but the results remain controversial and inconsistent. Therefore, we conducted a systematic review and meta-analysis of observational studies in order to precisely gauge the nature and magnitude of the association between NAFLD and risk of stroke. Given the high burden of NAFLD and stroke, we believe that clarification of this association might have important public health implications for the potential screening and management of patients with NAFLD and stroke.

## Methods

### Protocol and Registration

We conducted the systematic review and meta-analysis in accordance with the Preferred Reporting Items for Systematic Reviews and Meta-Analysis (PRISMA) statement (32). This study has been registered in advance on international prospective register of systematic reviews (PROSPERO) (CRD42020167330).

### Search Strategy

The PubMed, EMBASE and Web of Science were electrically searched from database inception until December 30, 2019, and updated on May 20, 2021. The search terms were as follow: (non-alcoholic fatty liver disease OR non-alcoholic fatty liver disease OR non-alcoholic fatty liver OR non-alcoholic fatty liver OR non-alcoholic steatohepatitis OR non-alcoholic steatohepatitis OR NAFLD OR NASH OR NAFL OR fatty liver) AND (stroke OR cerebral Infarction OR brain infarction OR cerebral hemorrhage OR intracerebral hemorrhage OR transient ischemic attack OR cerebrovascular disorders OR cerebrovascular accident). We used MeSH terms in combination with text word searching, without language restriction. Details of the search strategy for PubMed are presented in online Supplementary Table S1. Manual searches for additional studies were conducted by reviewing the reference lists of relevant studies to ensure completeness.

### Study Selection

Inclusion criteria were as follows: (1) Observational studies (i.e., cross-sectional, case-control, or cohort studies) investigating the risk of stroke among patients with NAFLD compared with individuals without NAFLD; (2) The diagnosis of NAFLD was based on liver histology, imaging (ultrasound, computed tomography, or magnetic resonance imaging), fatty liver index (FLI), or International Classification of Diseases (ICD) codes, in the absence of other causes of hepatic fat accumulation; (3) Reported adjusted or unadjusted estimates, i.e., odds ratio (OR), risk ratio (RR), hazard ratio (HR) with 95% confidence interval (CI), or the study provided adequate raw data to calculate them; (4) Based on data from the eligible studies, we also evaluated the relationship between “severe” NAFLD (NAFLD-fibrosis) and risk of stroke. The diagnosis of NAFLD-fibrosis was based on the NAFLD Fibrosis Score (NFS) or Fibrosis-4 score (FIB-4).

Exclusion criteria were as follows: (1) abstracts, comments, letters, case reports, laboratory studies, reviews and meta-analyses; (2) studies that used exclusively serum liver enzyme levels to diagnose NAFLD; (3) studies without comparators; (4) studies with insufficient data. When multiple studies using the same database/cohort or partially overlapping populations, the study with the largest sample size or the longest follow-up time was included. According to inclusion and exclusion criteria, two reviewers independently screened the studies by reading titles and abstracts, and obtained full texts of potentially relevant articles. Any disagreements were resolved by consensus.

### Data Extraction

A pre-designed data extraction form was utilized to collect information from eligible studies. We extracted the following data from each eligible study: the first author, publication year, study country, study design, data source (study subjects), study period, number of participants, mean age, methods used for diagnosing both NAFLD and stroke, follow-up time, OR, RR, HR (adjusted and unadjusted) with their 95%CI, and adjusted confounding variables. Two reviewers independently extracted the data from each selected study and any discrepancies were resolved by consensus.

### Quality Assessment and Grading the Strength of Evidence

The Newcastle-Ottawa Scale (NOS) (33) for case-control and cohort studies was used for methodological quality assessment. We used the modified NOS scale adapted for cross-sectional studies (34). NOS contains three major headings: selection, comparability, and exposure/outcome. A star system was used for study quality. The NOS assigns a maximum of four stars for selection (or five stars in cross-sectional studies), two stars for comparability and three stars for exposure/outcome. Studies were considered to be of high quality with the total score of 7 or higher, moderate quality with the total score of between 4 and 6, and <4 for low quality (35).

In addition, we used the GRADE (Grading of Recommendations, Assessment, Development and Evaluation) system to assess the quality of evidence of the outcomes in the included studies. The quality of evidence was categorized into four levels (i.e., high, moderate, low, and very low) in the GRADE system (36). As this study only included observational studies, which start with a “low quality” rating, may upgrade the quality of the evidence due to the following factors: large magnitude of effect, dose response relationship, and the effect plausible residual confounding factors. We used the GRADE profiler software (GradePro Version 3.6.1) to generate overall quality of evidence. Two reviewers independently appraised the methodological quality of each study and quality of evidence. Any discrepancies were resolved by consensus.

### Statistical Analysis

Statistical meta-analyses were conducted by the Review Manager software (Version 5.3, The Cochrane Collaboration, Copenhagen, Denmark). The pooled OR with 95% CI was combined by the generic inverse variance method of DerSimonian and Laird (37) based on a random-effects model. As the outcome of interest was relatively uncommon, we considered RR/HR equivalent to OR (38). In case of report of adjusted and unadjusted OR/RR/HR, the adjusted one for the most confounders was selected. Cochran's *Q*-test and *I*^2^ statistic were used to evaluate the statistic heterogeneity among studies. *P* < 0.10 for the *Q-*test was considered statistically significant. *I*^2^-values of 0–25%, 26–50%, 51–75%, and above 75% indicated insignificant, low, moderate, and high heterogeneity, respectively (39). When possible, subgroup analyses were further conducted to evaluate the influence of study design, study location, type of stroke, diagnostic methods of NAFLD, mean age of study participants, number of study participants sex, study quality, and adjustment for confounders on the pooled overall results and to explore potential sources of heterogeneity. Sensitivity analyses were conducted for the association by (1) excluding studies with fewer than 10,000 participants, (2) eliminating each of the included studies at a time. If the number of included studies ≥10 (40), publication bias was assessed by Begg's funnel plot (41) and Egger regression asymmetry test (42) using STATA/SE software (Version 12.0, STATA Corporation, Texas, USA), and *P* < 0.05 was considered statistically significant.

## Results

### Selection

**Figure 1:**
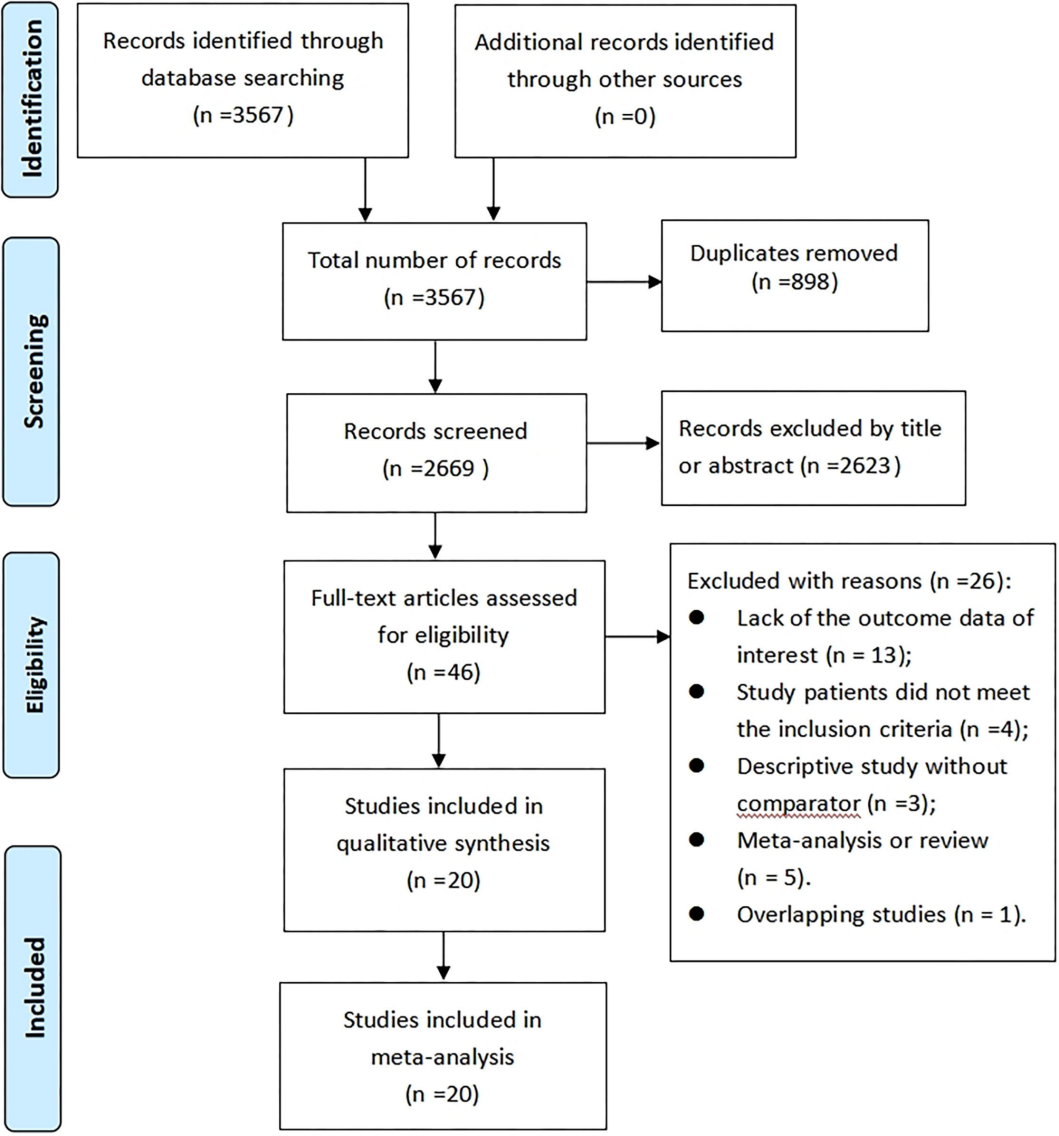
PRISMA flowchart of study selection process.

We initially identified 3,567 records from three electronic databases using the search strategy. Of these, 898 records were excluded because of duplicates. A total of 2,623 records were excluded by reviewing the title or abstract because the inclusion criteria were not met. Of remaining 46 articles, we further removed 26 articles by examining the full-text based on the selection criteria (description of excluded articles see Supplementary Table S2). As a result, 20 studies (12–31) were eligible for inclusion in this meta-analysis. Figure 1 displays the study screening process.

### Study Characteristics

The main characteristics of the included studies were summarized in Table 1. A total of 20 studies (involving 17,060,388 participants, 56% for male) have been published from 2007 to 2021. There were 14 cohort studies (12, 14, 16–18, 21–23, 25–29, 31), one case-cohort study (30), one case-control study (15), and four cross-sectional studies (13, 19, 20, 24). These studies came from various countries across four continents. Seven studies (13, 16, 18, 20, 22, 24, 30) were conducted in North America (United States), seven (12, 15, 19, 27–29, 31) in Asia (Japanese, Iran, Korea, China), five (17, 21, 23, 25, 26) in Europe (Italy, UK, Spain, Netherlands, Sweden, Germany), one (14) in Africa (Egypt). The sample size of included studies ranged widely, from 220 participants in an Iran case-control study to a study based on Korea nationwide health screening database of 95,84,399 participants. The mean age ranged between 48 and 67 years old, with years of follow up between 2.1 and 18.6 for cohort studies. With regard to the diagnosis of NAFLD, seven studies (12, 14, 15, 17, 19, 25, 31) used ultrasonography, four (18, 27–29) used fatty liver index, two studies (13, 23) used liver biopsy, two studies (16, 20) used computed tomography, while remaining three studies (21, 22, 26) used international classification of diseases (ICD) code to detect NAFLD. Three studies (24, 25, 30) used NAFLD fibrosis score (NFS) and Fibrosis-4 score (FIB-4) to diagnose NAFLD-fibrosis. Regarding type of stroke, most of studies did not define the subtypes of stroke, two studies (12, 14) contain different subtypes (ischemic stroke, cerebral hemorrhage). The diagnosis of stroke was mostly based on imaging, medical records, questionnaire and ICD code. The study continents, study subjects, follow-up time/study period, confounders adjustment and corresponding data of included studies are presented in Supplementary Table S3. The NOS scores of included studies ranged from 5 to 9 (mean 7.45). The details of methodological quality assessment of included studies with NOS were shown in Supplementary Table S4.

**Table 1:** Main characteristics of included studies.

**References**	**Country**	**Study design**	**Sample size**	**Mean age (years), male (%)**	**Diagnosis of NAFLD (NAFLD-fibrosis)**	**Type of stroke**	**Stroke verification**	**Nos. score**
Hamaguchi et al. (12)	Japanese	Cohort	1,221	48, 61.3%	USG	Ischemic stroke Hemorrhagic stroke	Physician diagnosed	6
Domanski et al. (13)	United States	Cross-sectional	377	54, 53.1%	Liver biopsy	Unspecific stroke	Medical records	6
El Azeem et al. (14)	Egypt	Cohort	747	51, 49%	USG	Ischemic stroke Hemorrhagic stroke	Medical records	5
Moshayedi et al. (15)	Iran	Case-control	220	66, 62.7%	USG	Ischemic stroke	CT and MRI	7
Pickhardt et al. (16)	United States	Cohort	1,050	51, 45.5%	CT	Unspecific stroke	Medical records	6
Fracanzani et al. (17)	Italy	Cohort	273	52, 87.2%	USG	Ischemic stroke	NR	7
Alexander et al. (18)	United States	Cohort	1,589	65, 45%	FLI (defined as FLI>60)	Unspecific stroke	Clinical evaluation	8
Kwak et al. (19)	Korea	Cross-sectional	1,014	50, 81.5%	USG	Ischemic stroke	MRI	8
Weinstein et al. (20)	United States	Cross-sectional	766	67, 46.5%	CT	Ischemic stroke	MRI	8
Alexander et al. (21)	UK, Italy, Spain, Netherlands	Cohort	4,751 086	55, 51%	ICD code	Unspecific stroke	ICD code	9
Allen et al. (22)	United States	Cohort	19,078	53, 47.6%	ICD and HICDA codes	Unspecific stroke	Diagnostic code	9
Hagstrom et al. (23)	Sweden	Cohort	1,493	48, 63%	Liver biopsy	Unspecific stroke	ICD code	8
Parikh et al. (24)	United States	Cross-sectional	27,040	57, 48.5%	NFS (> 0.676), FIB-4 (> 3.25)	Unspecific stroke	Self-reported (physician diagnosed)	7
Baratta et al. (25)	Italy	Cohort	898	56, 62.5%	USG, NFS (>0.676), FIB-4 (>2.67)	Ischemic stroke	Medical records	5
Labenz et al. (26)	Germany	Cohort	44,096	56, 50.2%	ICD code	Unspecific stroke	ICD code	8
Yang et al. (27)	Korea	Cohort	7,964	52, 41.6%	FLI (defined as FLI ≥60)	Unspecific stroke	Questionnaire	8
Lee et al. (28)	South Korea	Cohort	2,545,136	44, 62.8%	FLI (defined as FLI ≥ 60)	Unspecific stroke	ICD code	9
Lee et al. (29)	Korea	Cohort	9,584,399	50, 48.5%	FLI	Ischemic stroke	ICD code	8
Parikh et al. (30)	United States	Case-cohort	1,676	67, 43%	NFS (>0.676), FIB-4 (>3.25)	Ischemic stroke	ICD code	9
Xu et al. (31)	China	Cohort	79,905	52, 75.4%	USG	Ischemic stroke	ICD code	8

*NAFLD, non-alcoholic fatty liver disease; USG, Ultrasonography; CT, Computed tomography; MRI, Magnetic resonance imaging; NR, not reported; FLI, Fatty liver index; UK, United Kingdom; ICD, international classification of diseases; HICDA, Hospital International Classification of Diseases Adapted; NFS, The Non-alcoholic Fatty Liver Disease Fibrosis Score; FIB-4, Fibrosis-4 score; NOS, Newcastle-Ottawa Scale*.

### Association Between NAFLD and Risk of Stroke

**Figure 2:**
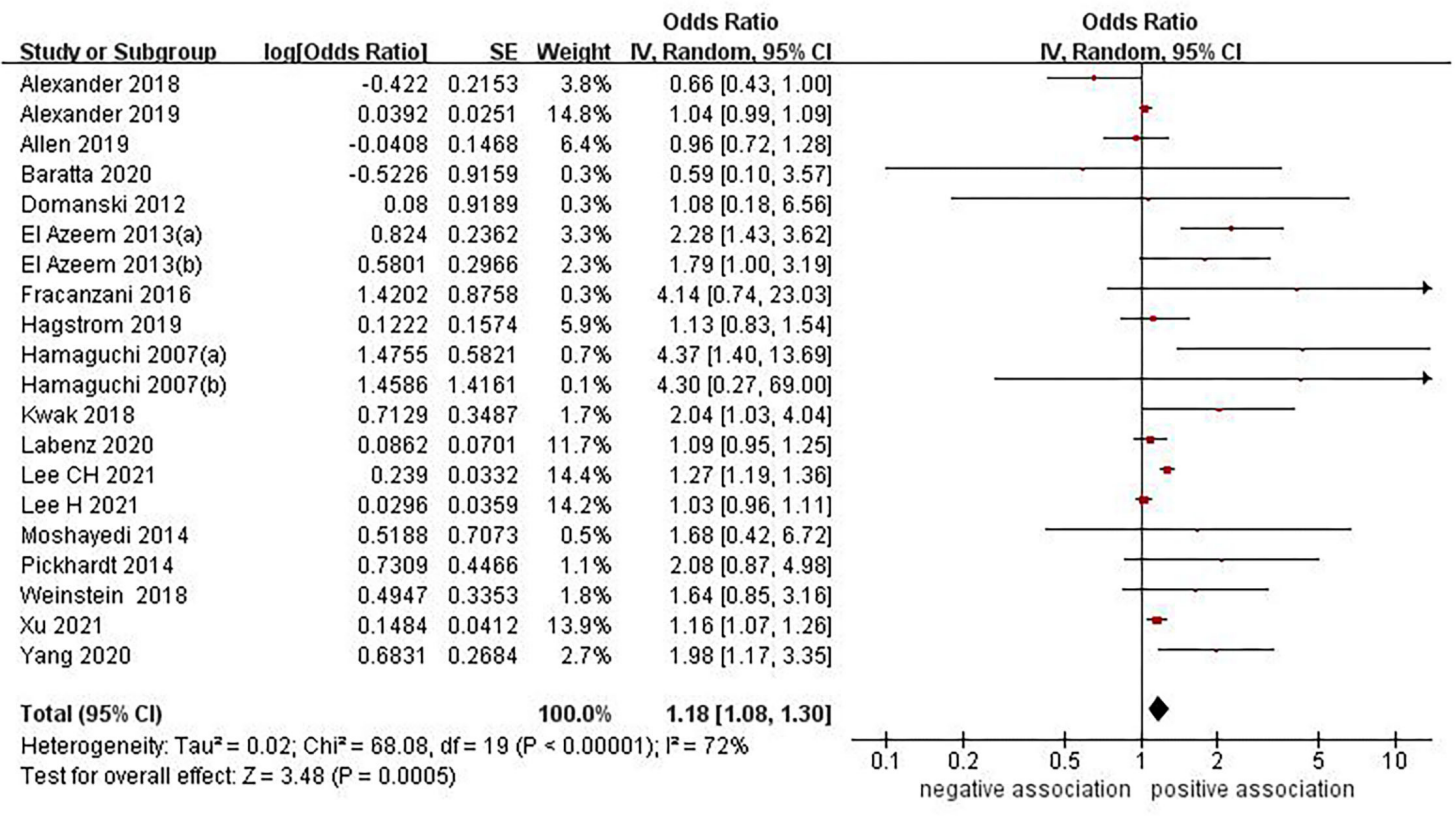
Forest plot of evaluating the association between NAFLD and stroke.

Eighteen studies (12–23, 25–29, 31) with 17,031,672 participants investigated the association between NAFLD and risk of stroke. On pooled analysis, NAFLD was significantly associated with increased risk of stroke (OR = 1.18, 95% CI: 1.08–1.30, *P* = 0.0005). Moderate heterogeneity was observed in the analysis (*I*^2^ = 72%, *P* < 0.00001) (Figure 2).

In view of the moderate heterogeneity, we performed numerous subgroup analyses. The concrete details of the subgroup analyses were presented in Table 2. In subgroup analysis stratified by study design, we found a positive association between NAFLD and stroke risk from 14 cohort studies (OR = 1.16, 95% CI: 1.06–1.28, *P* = 0.002; *I*^2^ = 76%) and 3 cross-sectional studies (OR = 1.76, 95% CI: 1.11–2.78, *P* = 0.02; *I*^2^ = 0%), respectively, whereas no significant association was observed in the subgroup of one case control study (OR = 1.68, 95% CI: 0.42–6.72, *P* = 0.01; *I*^2^ = 0%) (Supplementary Figure S1). In subgroup analysis stratified by study location, we found a positive association between NAFLD and stroke risk from Europe (OR = 1.05, 95% CI: 1.00–1.10, *P* = 0.04; *I*^2^ = 0%), Asia (OR = 1.24, 95% CI: 1.08–1.43, *P* = 0.002; *I*^2^ = 78%) and Africa (OR = 2.07, 95% CI: 1.44–2.98, *P* < 0.0001; *I*^2^ = 0%), respectively, whereas no significant association was observed in the subgroup of North America (OR = 1.07, 95% CI: 0.72–1.57, *P* = 0.75; *I*^2^ = 54%) (Supplementary Figure S2). In subgroup analysis stratified by type of stroke, we found that NAFLD was positively associated with the risk of both ischemic stroke (OR = 1.35, 95% CI: 1.11–1.63, *P* = 0.002; *I*^2^ = 71%) and hemorrhagic stroke (OR = 1.85, 95% CI: 1.05–3.27, *P* = 0.03; *I*^2^ = 0%), whereas no significant association was observed in the subgroup of unspecific stroke (OR = 1.11, 95% CI: 0.98–1.27, *P* = 0.10; *I*^2^ = 78%) (Supplementary Figure S3). In subgroup analysis stratified by diagnostic methods of NAFLD, we found a positive association when using the imaging techniques for diagnosing NAFLD (OR = 1.81, 95% CI: 1.31–2.49, *P* = 0.0003; *I*^2^ = 56%), whereas no significant association was observed when using diagnostic codes (OR = 1.04, 95% CI: 1.00–1.09, *P* = 0.07; *I*^2^ = 0%), FLI (OR = 1.12, 95% CI: 0.91–1.38, *P* = 0.29; *I*^2^ = 90%) or liver biopsy (OR = 1.13, 95% CI: 0.83–1.53, *P* = 0.44; *I*^2^ = 0%) for diagnosing NAFLD (Supplementary Figure S4). In subgroup analysis stratified by mean age of study participants, we found a positive association between NAFLD and stroke risk for studies with mean age <65 years old (OR = 1.20, 95% CI: 1.09–1.32, *P* = 0.0002; *I*^2^ = 75%). However, no significant association was observed for studies with mean age of more than or equal to 65 years old (OR = 1.09, 95% CI: 0.52–2.27, *P* = 0.81; *I*^2^ = 67%) (Supplementary Figure S5). We also conducted subgroup analyses based on number of study participants sex, study quality, and adjustment for confounders. The results of these subgroup analyses were consistent with the overall pooled results (Supplementary Figures S6–S8).

**Table 2:** Subgroup analyses of association between NAFLD and risk of stroke.

**Subgroup**	**No. of studies**	**OR (95%CI)**	***P*_association_**	***I^**2**^*(%)**	***P*_heterogeneity_**
**Overall studies**	18	1.18 (1.08–1.30)	0.0005	72	<0.00001
**Study design**						
Cohort	14	1.16 (1.06–1.28)	0.002	76	<0.00001
Case-control	1	1.68 (0.42–6.72)	0.46	-	-
Cross-sectional	3	1.76 (1.11–2.78)	0.02	0	0.78
**Study location**						
Europe	5	1.05 (1.00–1.10)	0.04	0	0.48
North America	5	1.07 (0.72–1.57)	0.75	54	0.07
Asia	7	1.24 (1.08–1.43)	0.002	78	<0.0001
Africa	1	2.07 (1.44–2.98)	<0.0001	0	0.52
**Type of stroke**						
Unspecific stroke	9	1.11 (0.98–1.27)	0.10	78	<0.0001
Ischemic stroke	9	1.35 (1.11–1.63)	0.002	71	0.0005
Hemorrhagic stroke	2	1.85 (1.05–3.27)	0.03	0	0.54
**Diagnostic methods of NAFLD**						
Imaging techniques	9	1.81 (1.31–2.49)	0.0003	56	0.01
Diagnostic codes	3	1.04 (1.00–1.09)	0.07	0	0.69
Fatty liver index	4	1.12 (0.91–1.38)	0.29	90	<0.00001
Liver biopsy	2	1.13 (0.83–1.53)	0.44	0	0.96
**Mean age**						
≥eanyears	3	1.09 (0.52–2.27)	0.81	67	0.05
<65 years	15	1.20 (1.09–1.32)	0.0002	75	<0.00001
**Number of sex**						
Male > female	11	1.17 (1.06–1.30)	0.003	70	0.0002
Male < female	7	1.32 (1.01–1.74)	0.04	76	0.0001
**Study quality**						
High	13	1.12 (1.03–1.23)	0.01	75	<0.00001
Moderate	5	2.09 (1.53–2.85)	<0.00001	0	0.58
**Adjustment for confounders**						
Adjusted	13	1.12 (1.03–1.23)	0.01	75	<0.00001
Unadjusted	11	1.62 (1.34–1.96)	<0.00001	67	0.0003

*OR, odds ratio; CI, confidence interval; NAFLD, non-alcoholic fatty liver disease*.

In order to confirm the robustness of the results, sensitivity analyses were conducted to assess whether excluding studies with fewer than 1,00,00 participants and eliminating each of the included studies at a time substantially altered the results of the remainders or not. We did not find significant changes in magnitude or direction of the summary estimates in all these conducted sensitivity analyses (Supplementary Table S5).

### Association Between NAFLD-Fibrosis and Risk of Stroke

**Figure 3:**
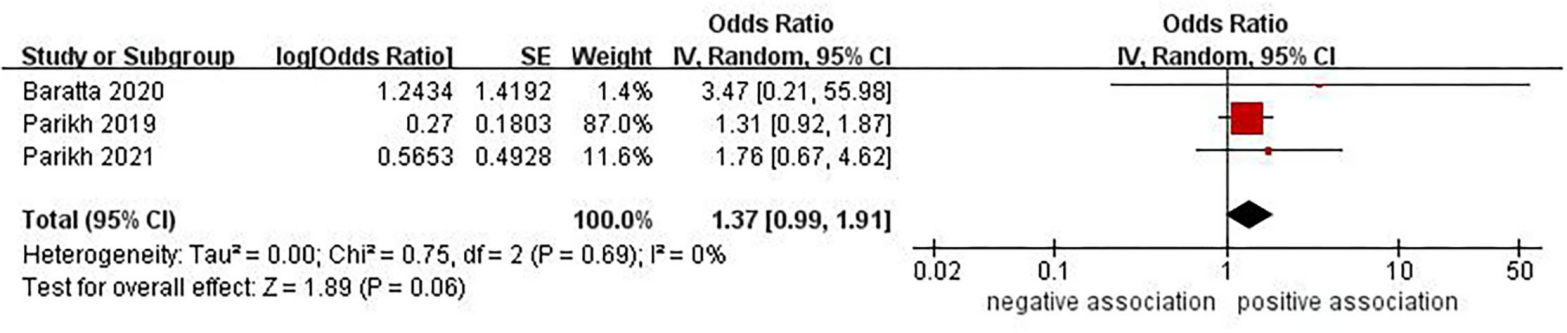
Forest plot of evaluating the association between NAFLD-fibrosis (by NFS) and stroke.

**Figure 4:**
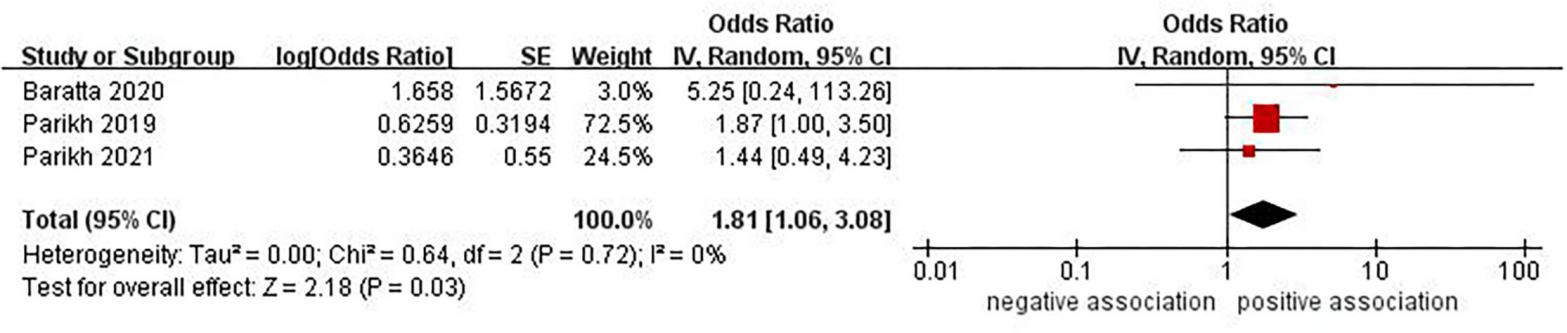
Forest plot of evaluating the association between NAFLD-fibrosis (by FIB-4) and stroke.

Three studies (24, 25, 30) with 29,614 participants investigated the association between NAFLD-fibrosis and risk of stroke. When NAFLD-fibrosis was defined using NFS, the pooled data showed that no significant association was observed (OR = 1.37, 95% CI: 0.99–1.91, *P* = 0.06) (Figure 3). No significant heterogeneity was observed in the analysis (*I*^2^ = 0%, *P* = 0.69). Sensitivity analysis did not alter the result. However, there was a positive correlation between NAFLD-fibrosis and risk of stroke (OR = 1.81, 95% CI: 1.06–3.08, *P* = 0.03) with insignificant heterogeneity (*I*^2^ = 0%, *P* = 0.72) when NAFLD-fibrosis was defined using FIB-4 (Figure 4). When we perform a sensitivity analysis by excluding the study by Parikh et al. (24), the result of this sensitivity analysis was contrary to the previous pooled result (OR = 1.66, 95% CI: 0.60–4.59, *P* = 0.33), indicating that the result was unstable.

### Grading the Strength of Evidence and Evaluation for Publication Bias

Owing to study design (observational studies only) and moderate heterogeneity, the GRADE assessment of the quality of the evidence was very low for both outcomes. The distribution of Begg's funnel plot for the association between NAFLD and risk of stroke was slightly asymmetrical by visual inspection (Supplementary Figure S9). Nevertheless, we used Egger's test to further quantitatively detect publication bias, indicating no evidence of substantive publication bias (*P*_egger_ = 0.063).

## Discussion

### Principal Findings

The aim of this current systematic review and meta-analysis was to synthesize the published literature on the association between NAFLD and the risk of stroke. To our best knowledge, the present study is the most current and largest meta-analysis on this topic to date. Meta-analysis of data from 18 studies with 17,031,672 participants has shown that NAFLD was associated with mildly increased risk of stroke (OR = 1.18, 95% CI: 1.08–1.30, *P* = 0.0005). Similar results were observed in most of the subgroup analyses we performed. Meanwhile, we have also investigated whether the severity of NAFLD (NAFLD-fibrosis) is associated with risk of stroke. Meta-analysis of data from 3 studies with 29,614 participants has shown that insufficient evidence to support the proposed association between NAFLD-fibrosis and an increased risk of stroke.

### Comparison With Previous Studies

In a previous smaller meta-analysis of three observational studies, Mahfood Haddad et al. (43) reported that patients with NAFLD had a significantly higher risk of ischemic stroke compared to the non-NAFLD group (fixed-effects RR: 2.09, 95% CI: 1.46–2.98, *P* < 0.001). In their meta-analysis, the authors included three prospective cohort studies (12, 14, 17) (published up to March 2016) that have also been incorporated into our meta-analysis. In 2018, a meta-analysis of seven observational studies (one cross-sectional, two case-control and four cohort studies) conducted by Hu et al. (44) showed that NAFLD was significantly associated with elevated risk of stroke (fixed-effects OR = 2.32, 95% CI 1.84–2.93, *P* < 0.001). Similar results were also observed when subgroup analyses were performed by ethnicity, study design and type of stroke. Compared with the meta-analysis conducted by Hu et al., our meta-analysis included almost all of their studies, with the exception of the study published by Ying et al. (45) (published in the form of a letter). Ying et al. (45) just relied on elevated serum alanine amino transferase (ALT) levels as a diagnostic tool of NAFLD, although serum ALT levels have been used to screen for NAFLD, levels of the marker may be normal in up to 79% of patients with established NAFLD (46), and elevated ALT levels do not provide sufficient information about the stage of NAFLD (i.e., NASH, fibrosis, cirrhosis), so the accuracy of their results might be questioned.

Compared to previous studies, the present meta-analysis advances the findings of this past work in different ways.

For one thing, our study covers the most comprehensive studies (*n* = 20 studies) and greatly increases the total sample size (*n* = 17,031,672 participants), especially by including most recent studies (14 updated studies) published between 2018 and 2021, providing newer and more sufficient epidemiologic evidence on the topic. For another, we further assessed the severity of NAFLD (NAFLD-fibrosis) and risk of stroke, although the number of studies is rare, firm conclusions have yet to be reached.

### Potential Explanations and Implications

The possible underlying mechanism for the development of stroke in patients with NAFLD remain poorly understood. There are few possible explanations. One possible explanation is that NAFLD promotes or accelerated the formation of atherosclerosis through insulin resistance, dyslipidemia, inflammatory response, oxidative stress, vasoactive and thrombogenic factors, gut-derived factors and mitochondrial dysfunction, as well as their interactions, which leads to the occurrence of stroke events (47–49). The other mechanism that could explain a possible association is that liver dysfunction caused by NAFLD can lead to thrombotic vascular disease by affecting the synthesis of coagulation protein, lipoprotein, and inflammation related factors (27).

The prevalence of NAFLD can vary by the ethnicity (1, 50). A previous study showed that Asians and Hispanics have a higher degree of steatosis than Whites and other ethnicities (51). There is research evidence that the association between NAFLD and increased inflammation remained significant for whites only, but not Chinese, Hispanics and African American after multivariable adjustment (52). Collectively, ethnicity may be a significant confounding factor when estimating the association between NAFLD and stroke risk. We conducted subgroup analysis based on different study locations to determine the risk of stroke related to NAFLD among different populations. The pooled results showed that NAFLD was associated with increased risk of stroke in European, Asian and African populations, but not in North American (United States). This may indicate that stroke risk assessment in Americans with NAFLD is important but should be done in the same way as for the general population. In view of the limited sample size of the American populations, prospective studies with large samples are needed in the future to further verify the relationship between NAFLD and stroke in the American populations. It is widely known that age and gender are important uncontrollable risk factors for stroke. In subgroup analysis stratified by mean age, we found that the risk of stroke was higher in patients with NAFLD patients under 65 years old. However, no significant association was observed for studies with mean age of more than or equal to 65 years old, which may be attributed to the insufficient statistical power because of the small sample.

We also conducted subgroup analyses based on number of study participants sex, the result showed that NAFLD could increase the risk of stroke regardless of number of study participants sex. Ischemic stroke and Hemorrhagic stroke are the main manifestations of stroke. Interestingly, subgroup analysis according to type of stroke found that NAFLD was associated with both ischemic and hemorrhagic stroke, but not with unspecific stroke. This may be attributed to the diversity of different stroke types in the same study might misestimate the risk. Further studies are needed to investigate the association between NAFLD and specific stroke types. With regard to the diagnostic methods of NAFLD, the result of our subgroup analysis showed that NAFLD diagnosed by imaging techniques (mainly ultrasonography) was associated with stroke, but NAFLD diagnosed by diagnostic codes, FLI, or liver biopsy was not significantly associated with stroke. This result showed that the risk of stroke was much stronger in patients with NAFLD diagnosed by imaging techniques (mainly ultrasonography). Ultrasonography is the most widely used non-invasive method for the diagnosis of NAFLD. Nevertheless, ultrasonography has inevitable limitations because of an incorrect diagnosis of NAFLD in 10–30% of patients (53), and cannot accurately diagnose NASH. Liver biopsy is the gold standard for clinical diagnosis of NAFLD, which is not suitable for epidemiological studies. FLI, a surrogate marker for NAFLD developed by Bedogni et al. (54). For fatty liver, FLI is not as accurate as liver biopsy or magnetic resonance imaging in the identification and grading of hepatic steatosis (55). Diagnostic codes may cause misclassification bias due to diagnostic miscoding or under coding (26). Most of the study participants in original studies used diagnostic codes or FLI to define NAFLD. Based on above considerations, the results should be interpreted with caution. In addition, there was no significant difference in studies with a case-control design, which may be due to the limited number of studies in these subgroups.

Given that NAFLD encompasses a spectrum of liver disease, these different stages of disease may have vastly different clinical outcomes. Stratified analysis of patients without the severity of NAFLD may restrict the conclusions (56). Therefore, we further analyzed the correlation between the severity of NAFLD and stroke. However, only three studies found insufficient evidence of a correlation between the severity of NAFLD (NAFLD-fibrosis) and stroke. Previously, a study conducted by Kim et al. (57) showed that liver fibrosis assessed with transient elastography was significantly associated with the risk of ischemic stroke. Recently, Xiong et al. (58), who included nine studies with 3,855,226 participants, showed that cirrhosis was associated with a higher risk of developing stroke, specifically hemorrhage stroke. These two studies suggest that severe stages of liver disease (fibrosis, cirrhosis) may be associated with an increased risk of stroke. However, our study reached the opposite conclusion, which may be due to the small sample leading to the insufficient statistical power. Future studies with a larger sample size are needed to verify the association between NAFLD-fibrosis and stroke.

These data provide a comprehensive insight into the association between NAFLD and risk of stroke based on the current evidence. We believe that the results of our meta-analysis are clinically relevant and further support the diagnosis of NAFLD, identifying a subset of individuals with a higher risk of sudden stroke, who need more rigorous monitoring and early treatment to potentially reduce the risk of stroke.

### Strengths and Limitations

The present study has several strengths. Firstly, as previously discussed, our study is the most current and largest meta-analysis to date aimed at investigating the association between NAFLD and the risk of stroke with a large sample size (17,060,388 participants). The large number of total cases provided high statistical power to quantitatively evaluate the association between NAFLD and stroke. Secondly, we conducted a comprehensive literature search, strict inclusion/exclusion criteria, rigorous quality assessment, and used GRADE system to assess the certainty of the evidence. And most of the studies included were of high quality, providing high-quality evidence for the topic. Thirdly, we performed a limited number of pre-planned subgroup analyses and comprehensive sensitivity analyses to further evaluate the correlation, which would contribute to understand the relationship more completely. All of these characteristics make our conclusions more reliable and convincing.

Notwithstanding these strengths, several limitations should be mentioned in our meta-analysis. First, our synthesis of the evidence was limited to observational studies, which are prone to confounders. Several studies did not report adjusted OR. Although most studies had adjusted for age, sex, hypertension, diabetes, dyslipidemia and smoking, lack of controlling for other known risk factors and potential confounding variables, such as physical activity, body mass index, alcohol consumption and personal history of cardiovascular disease. Furthermore, the metabolic syndrome and insulin resistance of the eligible studies reported incomplete adjustments, residual or unmeasured confounders cannot be excluded, which may affect the magnitude of the observed association and may lead to misleading overall results. Furthermore, prospective studies adjusting these confounders are needed to confirm the relationship. Second, regarding association between NAFLD and risk of stroke, the meta-analysis has a moderate heterogeneity (*I*^2^ = 72%) in the overall results, which was not explained by our sensitivity analyses. We conducted numerous preplanned subgroup analyses to assess the robustness of association and explore sources of heterogeneity. The heterogeneity may mainly come from different study designs, geographical location, type of stroke, diagnostic methods of NAFLD and adjustment of confounding factors. Third, data on the association between NAFLD severity (NASH, fibrosis or cirrhosis) and risk of stroke were derived from very few studies, and the use of non-invasive markers (such as the NFS and FIB-4) for NAFLD fibrosis has not been fully validated in the general population. Consequently, our current study lacks sufficient evidence regarding NAFLD severity (i.e., NASH) and risk of stroke. Fourth, although the results our subgroup analyses showed that NAFLD were more likely to be associated with stroke risk in patients under 65 years old, non-North American people and imaging diagnosed NAFLD, we did not have sufficient evidence to define different risk groups because of the limited sample sizes of some subgroups. Finally, the majority of study participants were conducted in European and Asia countries, with insufficient evidence from other regions. Whether the evidence can be directly extrapolated to other populations still needs further study. In spite of the limitations aforementioned, our results are robust enough to be considered valid and provide valuable updated evidence about the observed associations.

## Conclusions

In summary, we found that NAFLD was associated with increased risk of stroke. However, there was insufficient evidence to support the proposed association between NAFLD-fibrosis and an increased risk of stroke. To better understand any association, future well-designed prospective studies that take fully account of specific population, type of stroke, and confounding factors are warranted.

## Data Availability Statement

The original contributions presented in the study are included in the article/Supplementary Material, further inquiries can be directed to the corresponding authors.

## Author Contributions

B-GZ and MW performed the literature search, drafted the manuscript, and designed the systematic review. B-GZ and YZ screened the literature. X-FR, LL, and MW extracted data and assessed the quality. B-GZ, YZ, and BL analyzed and interpreted the data. MW, B-GZ, and Y-WA were responsible for the research design, data analysis, and manuscript revision. All authors gave their approval for the submission of the final manuscript.

## Funding

This work was supported by National Natural Science Foundation of China (No. 81774146).

## Conflict of Interest

The authors declare that the research was conducted in the absence of any commercial or financial relationships that could be construed as a potential conflict of interest.

## Publisher's Note

All claims expressed in this article are solely those of the authors and do not necessarily represent those of their affiliated organizations, or those of the publisher, the editors and the reviewers. Any product that may be evaluated in this article, or claim that may be made by its manufacturer, is not guaranteed or endorsed by the publisher.
