# Supplementary material for: Association Between Non-alcoholic Fatty Liver Disease and Risk of Stroke: A Systematic Review and Meta-Analysis
Source: Front Cardiovasc Med. 2022 Mar 8;9:812030. doi: 10.3389/fcvm.2022.812030 (PMC8957221; doi:10.3389/fcvm.2022.812030)
Supplement: Supplementary file 1 [file Data_Sheet_1.PDF]

Supplementary online material

Content

Content		Page
**Table S1.**	Representatives search strings for PubMed	2
**Table S2.**	Description of excluded studies	2-5
**Table S3.**	The other Characteristics of included studies	6-11
**Table S4.**	Methodological quality assessment of included studies with NOS	12
**Table S5.**	Results of sensitivity analyses	13
**Figure S1**.	Forest plot of subgroup analysis stratified by study design	14
**Figure S2**.	Forest plot of subgroup analysis stratified by study location	15
**Figure S3**.	Forest plot of subgroup analysis stratified by type of stroke	16
**Figure S4**.	Forest plot of subgroup analysis stratified by diagnostic methods of NAFLD	17
**Figure S5**.	Forest plot of subgroup analysis stratified by mean age of study participants	18
**Figure S6**.	Forest plot of subgroup analysis stratified by number of study participants sex	18
**Figure S7**.	Forest plot of subgroup analysis stratified by study quality	19
**Figure S8**.	Forest plot of subgroup analysis stratified by adjustment for confounders	20
**Figure S9**.	Begg's funnel plot for association between NAFLD and risk of stroke	21

Table S1. Representatives search strings for PubMed (from inception to 30 December 2019)	
**Databases**	**Search strings**
**PubMed**	("non-alcoholic fatty liver disease"[MeSH Terms] OR "non-alcoholic fatty liver disease"[tiab] OR "nonalcoholic fatty liver disease"[tiab] OR "non-alcoholic fatty liver"[tiab] OR "nonalcoholic fatty liver"[tiab] OR "nonalcoholic steatohepatitis"[tiab] OR "non-alcoholic steatohepatitis"[tiab] OR NAFLD[tiab] OR NASH[tiab] OR NAFL[tiab] OR "fatty liver"[MeSH Terms] OR "fatty liver"[tiab]) AND ("stroke"[MeSH Terms] OR "stroke"[tiab] OR "cerebral Infarction"[MeSH Terms] OR "cerebral Infarction"[tiab] OR "brain infarction"[MeSH Terms] OR "brain infarction"[tiab] OR "cerebral hemorrhage"[MeSH Terms] OR "cerebral haemorrhage"[tiab] OR "intracerebral haemorrhage"[tiab] OR "transient ischemic attack"[tiab] OR "cerebrovascular disorders"[MeSH Terms] OR "cerebrovascular disorders"[tiab] OR "cerebrovascular accident"[tiab])

Table S2. Description of excluded studies at the stage of eligibility according to the PRISMA flow chart.			
**No.**	**First author**	**Publication year**	**Reason for exclusion**
1.	Targher	2005	The study did not report the outcome of interest
2.	Tziomalos	2013	Descriptive study without comparator
3.	Pisto	2014	Study patients did not meet the inclusion criteria
4.	Mantovani	2016	The study did not report the outcome of interest
5.	Abdeldyem	2017	Descriptive study without comparator
6.	Simon	2017	The study did not report the outcome of interest
7.	Alkagiet	2018	Review
8.	Hu	2018	Meta-analysis

9.	Lee	2018	The study did not report the outcome of interest
10.	Li	2018	The study did not report the outcome of interest
11.	Sao	2018	Review
12.	Wójcik-Cichy	2018	Review
13.	Baik	2019	Study patients did not meet the inclusion criteria
14.	Golabi	2019	The study did not report the outcome of interest
15.	Jang	2019	The study did not report the outcome of interest
16.	Lin	2019	Study patients did not meet the inclusion criteria
17.	Liu	2019	The study did not report the outcome of interest
18.	Moretti	2019	The study did not report the outcome of interest
19.	Tu	2019	Descriptive study without comparator
20.	Weinstein	2019	The study did not report the outcome of interest
21.	Henson	2020	Study patients did not meet the inclusion criteria
22.	Kim	2020	Overlapping studies, it was from the same database conducted by Lee H et al (2021) , but the sample size and follow-up time of the study were smaller than that of the study
23.	Parikh	2020	The study did not report the outcome of interest
24.	Pastori	2021	The study did not report the outcome of interest
25.	Veracruz	2021	Review
26.	Seo	2021	The study did not report the outcome data of interest

References for the table

1. Targher G, Bertolini L, Poli F, et al. Nonalcoholic fatty liver disease and risk of future cardiovascular events among type 2 diabetic patients.Diabetes. 2005; 54: 3541-3546.

2. Tziomalos K, Giampatzis V, Bouziana SD, et al. Association between nonalcoholic fatty liver disease and acute ischemic stroke severity and outcome. World J Hepatol. 2013; 5: 621-626.

3. Pisto P, Santaniemi M, Bloigu R, et al. Fatty liver predicts the risk for cardiovascular events in middle-aged population: a population-based cohort study. BMJ Open. 2014;4:e004973.

4. Mantovani A, Mingolla L, Rigolon R, et al. Nonalcoholic fatty liver disease is independently associated with

an increased incidence of cardiovascular disease in adult patients with type 1 diabetes. International Journal of Cardiology. 2016;225:387-391.

5. Abdeldyem SM, Goda T, Khodeir SA, Abou Saif S, Abd-Elsalam S. Nonalcoholic fatty liver disease in patients with acute ischemic stroke is associated with more severe stroke and worse outcome. Journal of Clinical Lipidology. 2017;11:915-919.

6. Simon TG, Kartoun U, Zheng H, et al. MELD-Na score predicts incident major cardiovascular events, in patients with nonalcoholic fatty liver disease. Hepatol Commun. 2017;1:429-438.

7. Alkagiet S, Papagiannis A, Tziomalos K. Associations between nonalcoholic fatty liver disease and ischemic stroke. World journal of hepatology. Jul 27 2018;10:474-478.

8. Hu J, Xu Y, He Z, et al. Increased risk of cerebrovascular accident related to non-alcoholic fatty liver disease: A meta-analysis. Oncotarget. 2018;9:2752-2760.

9. Lee JE, Lee YJ, Chung SY, Cho HW, Park BJ, Jung DH. Severity of nonalcoholic fatty liver disease is associated with subclinical cerebro-cardiovascular atherosclerosis risk in Korean men. PLoS ONE. 2018;13: e0193191.

10. Li H, Hu B, Wei L, et al. Non-alcoholic fatty liver disease is associated with stroke severity and progression of brainstem infarctions. European Journal of Neurology. 2018;25:577-e34.

11. Sao R, Aronow WS. Association of non-alcoholic fatty liver disease with cardiovascular disease and subclinical atherosclerosis. Archives of Medical Science. 2018;14:1233-1244.

12. Wójcik-Cichy K, Koślińska-Berkan E, Piekarska A. The influence of NAFLD on the risk of atherosclerosis and cardiovascular diseases. Clinical and Experimental Hepatology. 2018;4:1-6.

13. Baik M, Kim SU, Kang S, et al. Liver Fibrosis, Not Steatosis, Associates with Long-Term Outcomes in Ischaemic Stroke Patients. Cerebrovascular Diseases. 2019;47:32-39.

14. Golabi P, Paik J, Fukui N, Locklear CT, de Avilla L, Younossi ZM. Patients with lean nonalcoholic fatty liver disease are metabolically abnormal and have a higher risk for mortality. Clinical Diabetes. 2019;37:65-72.

15. Jang H, Kang D, Chang Y, et al. Non-alcoholic fatty liver disease and cerebral small vessel disease in Korean cognitively normal individuals. Scientific reports. 2019;9:1814.

16. Lin S-Y, Lin C-L, Chen W-S, et al. Association Between Alcoholic Cirrhosis and Hemorrhagic Stroke: A Nationwide Population-based Study. Alcohol and Alcoholism. May 2019;54:302-309.

17. Liu H-H, Cao Y-X, Sun D, et al. Impact of Non-Alcoholic Fatty Liver Disease on Cardiovascular Outcomes in Patients With Stable Coronary Artery Disease: A Matched Case-Control Study. Clinical and Translational Gastroenterology. 2019;10:e00011.

18. Moretti R, Caruso P, Gazzin S. Non-alcoholic fatty liver disease and neurological defects. Annals of Hepatology. 2019;18:563-570.

19. Tu S, Zhao R, Fang H, Wang L, Shao A, Sheng J. Association between Non-Alcoholic Fatty Liver Disease and Intracerebral Hemorrhage. Cell Transplantation. 2019;28:1033-1038.

20. Weinstein G, Davis-Plourde K, Himali JJ, Zelber-Sagi S, Beiser AS, Seshadri S. Non-alcoholic fatty liver

disease, liver fibrosis score and cognitive function in middle-aged adults: The Framingham Study. Liver International. 2019;39:1713-1721.

21. Henson JB, Simon TG, Kaplan A, Osganian S, Masia R, Corey KE. Advanced fibrosis is associated with incident cardiovascular disease in patients with non-alcoholic fatty liver disease. *Aliment Pharmacol Ther*. 2020;51: 728-736.

22. Kim JH, Moon JS, Byun SJ, Lee JH, Kang DR, Sung KC,et al. Fatty liver index and development of cardiovascular disease in Koreans without pre-existing myocardial infarction and ischemic stroke: a large population-based study. Cardiovasc Diabetol. 2020;19(1):51.

23. Parikh NS, Kamel H, Navi BB, Iadecola C, Merkler AE, Jesudian A, Dawson J, et al. Liver fibrosis indices and outcomes after primary intracerebral hemorrhage. Stroke. 2020;51:830-837.

24. Pastori D, Sciacqua A, Marcucci R, Del Ben M, Baratta F, Violi F, et al. Non-alcoholic fatty liver disease (NAFLD), metabolic syndrome and cardiovascular events in atrial fibrillation. A prospective multicenter cohort study. Internal and emergency medicine. 2021. doi: 10.1007/s11739-021-02682-3.Online ahead of print.

25. Veracruz N, Hameed B, Saab S, Wong RJ. The Association Between Nonalcoholic Fatty Liver Disease and Risk of Cardiovascular Disease, Stroke, and Extrahepatic Cancers. Journal of clinical and experimental hepatology. 2021;11:45-81.

26. Seo BS, Roh JH, Lee JH, Lee H, Min Kim Y, Yoon YH, et al. Association between nonalcoholic fatty liver disease and cardiovascular disease revealed after comprehensive control of metabolic risk factors: a nationwide population-based study in Korea. Eur J Gastroenterol Hepatol. 2021. Epub 2021/03/19. doi: 10.1097/meg.0000000000002102.

Table S3 The Characteristics of included studies about continents, study subjects, follow-up time (years)/study period, confounders adjustment and corresponding data in the meta-analysis					
**First author, year**	**Continents**	**Study subjects**	**Follow-up time (years)/ Study period**	**Confounders adjustment**	**OR/HR(95%CI)**
Hamaguchi, 2007	Asia	Subjects with medical health checkup program at Murakami Memorial Hospital, Gifu, Japan	5	None	cOR:<br>IS: 4.37 (1.40-13.69)<br>CH: 4.30 (0.27-69.00)
Domanski, 2012	North America	Patients who underwent liver biopsies at the Brooke Army Medical Center within the Gastroenterology and Hepatology clinic.	January 2003 to December 2009	None	cOR: 1.08 (0.18-6.56)
El Azeem, 2013	Africa	Subjects with normal or near normal and kidney functions, and without overt protienuria or history of cardiovascular events between January 2009 and February 2010	3	None	cOR:<br>IS: 2.28 (1.43-3.62)<br>CH: 1.79 (1.00-3.19)
Moshayedi, 2014	Asia	Patients with stroke were evaluated by CT scan and MRI from Razi and Imam Reza University Hospitals of Tabriz University of Medical Science, Tabriz, Iran. Controls include age and sex matched individuals without history of stroke from the same institution.	May 2012 to November 2013	Age, sex,waist circumference, hypertension, triglyceride, DM, LDL , ALT, AST, BMI, Creatine, cigarette smoking, and ischemic heart disease.	cOR: 2.54 (1.41-4.55)<br>aOR: 1.68 (0.42-6.76)

Pickhardt, 2014	North America	Consecutive unenhanced abdominal CT studies in adults within PACS at the University of Wisconsin Health Sciences from March 2001 to February 2002.	7.5 (mean)	None	cOR: 2.08 (0.87-4.98)
Fracanzani, 2016	Europe	Italian patients with NAFLD and age- ,sex- and BMI-matched control individuals without known liver diseases at the Center for the Study of Atherosclerosis, University of Milan.	10	Age, sex, BMI	aOR: 4.14 (0.74-23.03)
Alexander, 2018	North America	The REasons for Geographic and Racial Differences in Stroke (REGARDS) cohort study patients were recruited from 2003–2007. REGARDS consisting of 572 cases of incident ischemic stroke and a stratified cohort random sample of 1,017 participants.	5.8 (mean)	Age, race, age*race, SBP, left ventricular hypertrophy, smoking, prevalent CVD, atrial fibrillation, DM, and hypertension medication use	aHR:<br>All: 0.65 (0.43-1.00)<br>Male: 0.50 (0.26-0.96)<br>Female: 1.03 (0.58-1.82)
Kwak, 2018	Asia	Subjects who underwent brain MRI and abdominal ultrasonography (US) were recruited during a voluntary health screening program at the Seoul National University Hospital Gangnam Center between 2007 and 2009	2007 to 2009	Age, sex, smoking status, alcohol consumption, diabetes and hypertension	aOR: 2.04 (1.03-4.03)

Weinstein, 2018	North America	Participants were recruited from the Offspring cohort of the Framingham Study.	November 2002 to April 2005	Age at MRI, age-squared at MRI, sex, alcohol (drinks per week), years between CT scan and covariate assessment, years between CT scan and MRI, visceral adipose tissue, BMI, SBP, hypertension, levels for HDL-C and LDL-C, lipid treatment, current smoking, DM, history of CVD, physical activity index, homeostatic model assessment of insulin resistance, and levels for CRP and homocysteine.	cOR: 1.09 (0.65-1.85)<br>aOR: 1.64 (0.85-3.16)
Alexander, 2019	Europe	Patients data were included from four primary care databases available through the EMIF network: The Health Improvement Network (THIN, UK), Health Search Database (HSD, Italy), Information System for Research in Primary Care (SIDIAP, Spain), and Integrated Primary Care Information (IPCI,Netherlands).	2.1-5.5 (mean)	Age, smoking status, DM, SBP, total cholesterol level, statin use, and hypertension.	aHR: 1.04 (0.99-1.09)

Allen, 2019	North America	All adult individuals diagnosed with NAFLD in Olmsted County, MN, between 1997 and 2014, using prospectively collected data in a medical record linkage system, the Rochester Epidemiology Project (REP).	7 (mean)	Age, sex, personal history of CVD, BMI, time-dependent smoking, DM, hypertension, and dyslipidemia.	cOR:1.31 (1.12-1.53)<br>aHR: 0.96 (0.72-1.26)
Hagstrom, 2019	Europe	Patients with biopsy-proven NAFLD at Linköping University Hospital and the KarolinskaUniversity Hospital, Huddinge during 1971 to 2009.	18.6 (mean)	Age, sex and municipality	cOR:1.01 (0.74-1.36)<br>aHR: 1.13(0.83-1.54)
Parikh, 2019	North America	Adult participants 21 years of age and older enrolled in National Health and Nutrition Examination Survey (NHANES) data from 2005 to 2014.	2005 to 2014	Age, sex, race/ethnicity, insurance status, poverty, education, physical inactivity, hypertension, diabetes, dyslipidemia, smoking, and obesity.	aOR:<br>All: 1.35(0.96-1.90)<br>NFS: 1.31(0.92-1.87)<br>FIB-4: 1.87(1.00-3.50)
Baratta, 2020	Europe	898 consecutive outpatients referred to the Day Service of Internal Medicine and Metabolic Disorders of the Policlinico Umberto I University Hospital in Rome with at least one out of the following cardio-metabolic diseases: arterial hypertension, overweight /obesity,T2DM, dyslipidaemia, atrial fibrillation, metabolic syndrome.	3.5 (mean)	None	cOR:<br>All: 0.59(0.10-3.57)<br>NFS:3.47(0.21-55.97)<br>FIB-4:5.25(0.24-113.25)

Labenz, 2020	Europe	Adult patients (≥ 18 years) with an initial diagnosis of NAFLD/NASH in 1,262 general practices in Germany between January 2000 and December 2015.	10	Age, sex, treating physician, index year, DM, hypertension, and hyperlipidemia.	aHR:<br>All: 1.09 (0.95-1.24)<br>Male: 0.99 (0.82-1.19)<br>Female: 1.21 (0.99-1.48)
Yang, 2020	Asia	Adult participants aged 40 to 69 from Ansung–Ansan cohort	12	Sex, age,hypertension, DM, hyperlipidemia, CVD,smoking and alcohol status, and BMI, metabolic equivalent of task, cancer, hyperlipidemia drug, and antihypertension drug.	cOR: 1.92 (1.29-2.85)<br>aHR: 1.98 (1.17-3.34)
Lee CH, 2021	Asia	Participants older than 20 years who had undergone four Korean Health Screening examinations from 2009 until 2013 were initially included.	5.1 (mean)	Age, sex, smoking, drinking, income, hypertension, dyslipidemia, diabetes, BMI, and regular physical activity.	cOR: 1.78 (1.68-1.89)<br>aHR: 1.27(1.19-1.37)
Lee H, 2021	Asia	Adults 40–64 years of age who underwent routine National Health Insurance Service (NHIS) health examinations in 2009–2010.	10.1 (mean)	Age, sex, household income quartile, residential area, Charlson Comorbidity Index, tobacco use, exercise frequency, and estimated glomerular filtration rate.	aHR: 1.03 (0.96-1.12)
Parikh, 2021	North America	Black and white participants recruited from 2003 to 2007 from the contiguous United States and followed prospectively for stroke.	5.4 (mean)	Age, sex, and race,hypertension, SBP, diabetes, current smoking, atrial fibrillation, left ventricular hypertrophy, and baseline CVD,	aHR:<br>NFS: 1.76 (0.67, 4.61)<br>FIB-4: 1.44 (0.49, 4.28)

				aspirin and warfarin use.	
Xu, 2021	Asia	Active and retired employees aged ≥18 years of the Kailuan Group, Tangshan, China, were invited to participate in examination in 2006.	10.34 (mean)	Age, sex, physical activity, BMI, smoker, history of hypertension, diabetes, atrial fibrillation, hypercholesterolemia, lipid-lowering medication, HDL, triglyceride, hsCRP, and fasting blood glucose	aHR: 1.16 (1.07 – 1.26)

CT, computed tomography; MRI, magnetic resonance imaging; DM, diabetes mellitus; LDL, Low-density lipoprotein; ALT, alanine aminotransferase; AST, aspartate aminotransferase; BMI, body mass index; PACS, Picture Archiving and Communication system; NAFLD, nonalcoholic fatty liver disease; SBP, systolic blood pressure; CVD, cardiovascular disease; HDL-C, high-density lipoprotein cholesterol; LDL-C, low-density lipoprotein cholesterol; CRP, C-reactive protein; NASH, nonalcoholic steatohepatitis;NFS, The Nonalcoholic Fatty Liver Disease Fibrosis Score; FIB-4, Fibrosis-4 score; OR, odds ratio; HR, hazard ratio; CI, confidence interval; cOR, crude OR; aOR, adjusted OR; aHR, adjusted HR; IS, Ischemic stroke; CH, Cerebral hemorrhage.

Table S4 Methodological quality assessment of included studies with NOS.

First author, year	Selection	Comparability	Exposure/Outcome	Total (0-9)	Quality
Hamaguchi, 2007	✸✸✸✸	–	✸✸	6	Moderate
Domanski, 2012	✸✸✸✸	–	✸✸	6	Moderate
El Azeem, 2013	✸✸✸✸	–	✸	5	Moderate
Moshayedi, 2014	✸✸✸	✸✸	✸✸	7	High
Pickhardt, 2014	✸✸✸✸	–	✸✸	6	Moderate
Fracanzani, 2016	✸✸✸	✸✸	✸✸	7	High
Alexander, 2018	✸✸✸✸	✸✸	✸✸	8	High
Kwak, 2018	✸✸✸	✸✸	✸✸✸	8	High
Weinstein, 2018	✸✸✸	✸✸	✸✸✸	8	High
Alexander, 2019	✸✸✸✸	✸✸	✸✸✸	9	High
Allen, 2019	✸✸✸✸	✸✸	✸✸✸	9	High
Hagstrom, 2019	✸✸✸✸	✸–	✸✸✸	8	High
Parikh, 2019	✸✸✸	✸✸	✸✸	7	High
Baratta, 2020	✸✸✸	–	✸✸	5	Moderate
Labenz, 2020	✸✸✸✸	✸✸	✸✸	8	High
Yang, 2020	✸✸✸	✸✸	✸✸✸	8	High
Lee CH, 2021	✸✸✸✸	✸✸	✸✸✸	9	High
Lee H, 2021	✸✸✸	✸✸	✸✸✸	8	High
Parikh, 2021	✸✸✸✸	✸✸	✸✸✸	9	High
Xu, 2021	✸✸✸	✸✸	✸✸✸	8	High

Note: NOS, Newcastle–Ottawa Scale; "- "stands for zero point.

Table S5   Results of sensitivity analyses

**Studies omitted**	**OR (95% CI)**	$P_{association}$	**Heterogeneity**
＜10000 participants	1.10 (1.02-1.20)	0.02	I^2 = 83%, P ＜0.0001
Hamaguchi (2007)	1.17 (1.07-1.28)	0.001	I^2 = 72%, P ＜0.00001
Domanski (2012)	1.18 (1.08-1.30)	0.0005	I^2 = 74%, P ＜0.00001
El Azeem (2013)	1.14 (1.04-1.25)	0.004	I^2 = 70%, P ＜0.00001
Moshayedi (2014)	1.18 (1.07-1.30)	0.0006	I^2 = 73%, P ＜0.00001
Pickhardt (2014)	1.18 (1.07-1.29)	0.0008	I^2 = 73%, P ＜0.00001
Fracanzani (2016)	1.18 (1.07-1.29)	0.0007	I^2 = 73%, P ＜0.00001
Alexander (2018)	1.21 (1.10-1.32)	＜0.0001	I^2 = 71%, P ＜0.00001
Kwak (2018)	1.17 (1.07-1.29)	0.001	I^2 = 72%, P ＜0.00001
Weinstein (2018)	1.18 (1.07-1.29)	0.0009	I^2 = 73%, P ＜0.00001
Alexander (2019)	1.22 (1.09-1.37)	0.0004	I^2 = 68%, P ＜0.00001
Allen (2019)	1.20 (1.09-1.33)	0.0003	I^2 = 73%, P ＜0.00001
Hagstrom (2019)	1.19 (1.08-1.31)	0.0006	I^2 = 74%, P ＜0.00001
Baratta (2020)	1.19 (1.08-1.31)	0.0005	I^2 = 73%, P ＜0.00001
Labenz (2020)	1.20 (1.08-1.33)	0.0006	I^2 = 74%, P ＜0.00001
Yang (2020)	1.16 (1.06-1.28)	0.001	I^2 = 72%, P ＜0.00001
Lee CH (2021)	1.16 (1.05-1.28)	0.003	I^2 = 62%, P =0.0002
Lee H (2021)	1.22 (1.09-1.36)	0.0004	I^2 = 71%, P ＜0.00001
Xu (2021)	1.20 (1.07-1.34)	0.001	I^2 = 73%, P ＜0.00001

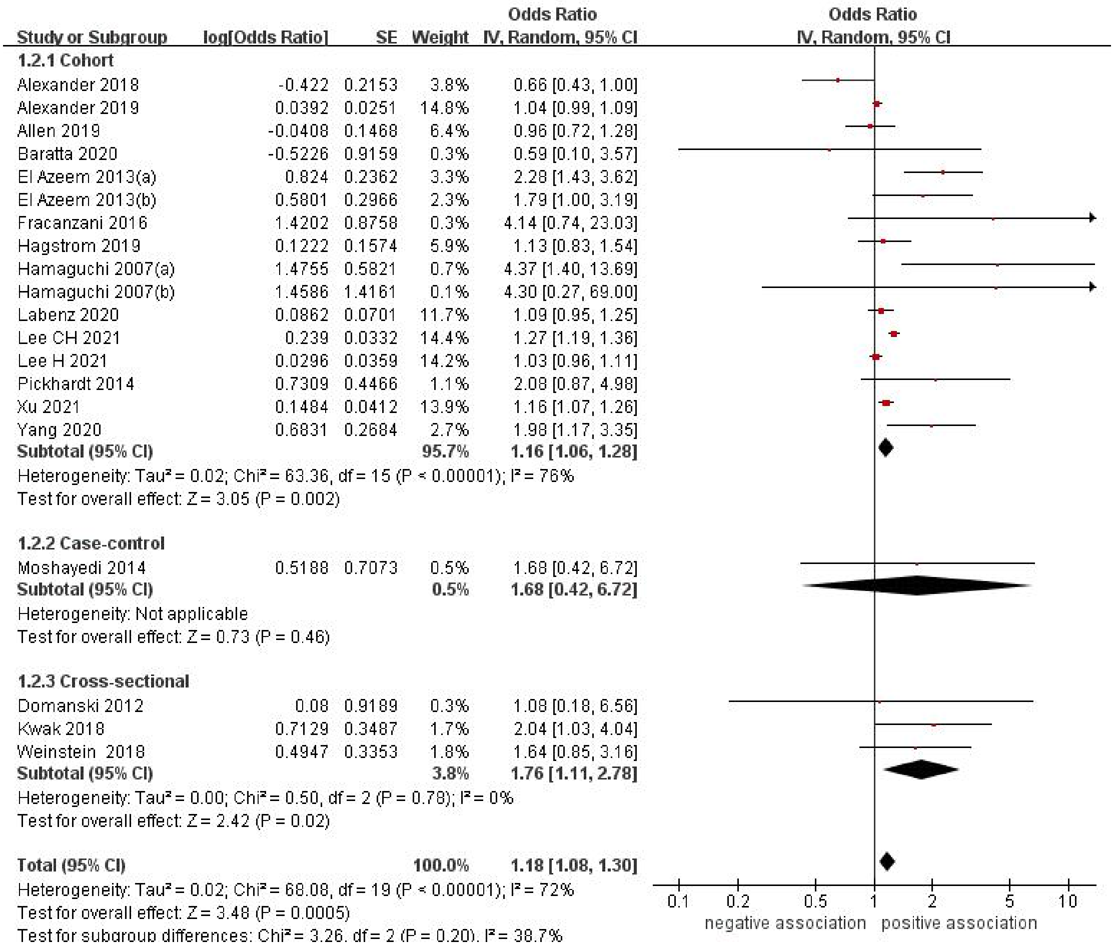

Figure S1. Forest plot of subgroup analysis stratified by study design

Study or Subgroup	log[Odds Ratio]	SE	Weight	Odds Ratio IV, Random, 95% CI
**1.3.1 Europe**				
Alexander 2019	0.0392	0.0251	14.8%	1.04 [0.99, 1.09]
Baratta 2020	-0.5226	0.9159	0.3%	0.59 [0.10, 3.57]
Fracanzani 2016	1.4202	0.8758	0.3%	4.14 [0.74, 23.03]
Hagstrom 2019	0.1222	0.1574	5.9%	1.13 [0.83, 1.54]
Labenz 2020	0.0862	0.0701	11.7%	1.09 [0.95, 1.25]
**Subtotal (95% CI)**			**32.9%**	**1.05 [1.00, 1.10]**
Heterogeneity: Tau² = 0.00; Chi² = 3.48, df = 4 (P = 0.48); I² = 0%				
Test for overall effect: Z = 2.01 (P = 0.04)				
**1.3.2 North America**				
Alexander 2018	-0.422	0.2153	3.8%	0.66 [0.43, 1.00]
Allen 2019	-0.0408	0.1468	6.4%	0.96 [0.72, 1.28]
Domanski 2012	0.08	0.9189	0.3%	1.08 [0.18, 6.56]
Pickhardt 2014	0.7309	0.4466	1.1%	2.08 [0.87, 4.98]
Weinstein 2018	0.4947	0.3353	1.8%	1.64 [0.85, 3.16]
**Subtotal (95% CI)**			**13.4%**	**1.07 [0.72, 1.57]**
Heterogeneity: Tau² = 0.09; Chi² = 8.68, df = 4 (P = 0.07); I² = 54%				
Test for overall effect: Z = 0.32 (P = 0.75)				
**1.3.3 Asia**				
Hamaguchi 2007(a)	1.4755	0.5821	0.7%	4.37 [1.40, 13.69]
Hamaguchi 2007(b)	1.4586	1.4161	0.1%	4.30 [0.27, 69.00]
Kwak 2018	0.7129	0.3487	1.7%	2.04 [1.03, 4.04]
Lee CH 2021	0.239	0.0332	14.4%	1.27 [1.19, 1.36]
Lee H 2021	0.0296	0.0359	14.2%	1.03 [0.96, 1.11]
Moshayedi 2014	0.5188	0.7073	0.5%	1.68 [0.42, 6.72]
Xu 2021	0.1484	0.0412	13.9%	1.16 [1.07, 1.26]
Yang 2020	0.6831	0.2684	2.7%	1.98 [1.17, 3.35]
**Subtotal (95% CI)**			**48.1%**	**1.24 [1.08, 1.43]**
Heterogeneity: Tau² = 0.02; Chi² = 31.29, df = 7 (P < 0.0001); I² = 78%				
Test for overall effect: Z = 3.06 (P = 0.002)				
**1.3.4 Africa**				
El Azeem 2013(a)	0.824	0.2362	3.3%	2.28 [1.43, 3.62]
El Azeem 2013(b)	0.5801	0.2966	2.3%	1.79 [1.00, 3.19]
**Subtotal (95% CI)**			**5.6%**	**2.07 [1.44, 2.98]**
Heterogeneity: Tau² = 0.00; Chi² = 0.41, df = 1 (P = 0.52); I² = 0%				
Test for overall effect: Z = 3.95 (P < 0.0001)				
**Total (95% CI)**			**100.0%**	**1.18 [1.08, 1.30]**
Heterogeneity: Tau² = 0.02; Chi² = 68.08, df = 19 (P < 0.00001); I² = 72%				
Test for overall effect: Z = 3.48 (P = 0.0005)				
Test for subgroup differences: Chi² = 18.02, df = 3 (P = 0.0004), I² = 83.3%				

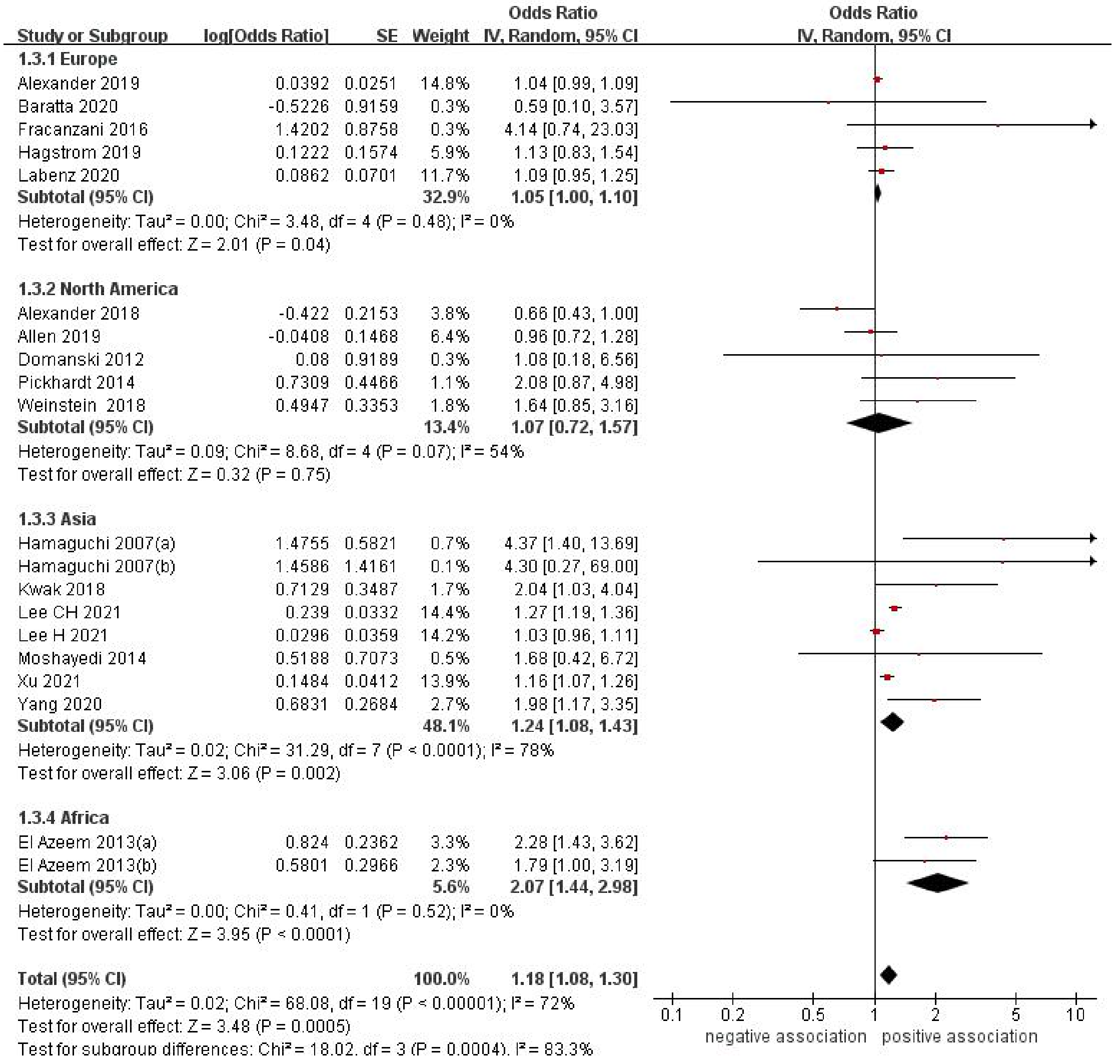

Figure S2. Forest plot of subgroup analysis stratified by study location

Study or Subgroup	log[Odds Ratio]	SE	Weight	Odds Ratio IV, Random, 95% CI
**1.4.1 Unspecific stroke**				
Alexander 2018	-0.422	0.2153	3.8%	0.66 [0.43, 1.00]
Alexander 2019	0.0392	0.0251	14.8%	1.04 [0.99, 1.09]
Allen 2019	-0.0408	0.1468	6.4%	0.96 [0.72, 1.28]
Domanski 2012	0.08	0.9189	0.3%	1.08 [0.18, 6.56]
Hagstrom 2019	0.1222	0.1574	5.9%	1.13 [0.83, 1.54]
Labenz 2020	0.0862	0.0701	11.7%	1.09 [0.95, 1.25]
Lee CH 2021	0.239	0.0332	14.4%	1.27 [1.19, 1.36]
Pickhardt 2014	0.7309	0.4466	1.1%	2.08 [0.87, 4.98]
Yang 2020	0.6831	0.2684	2.7%	1.98 [1.17, 3.35]
**Subtotal (95% CI)**			**61.0%**	**1.11 [0.98, 1.27]**
Heterogeneity: Tau² = 0.02; Chi² = 36.81, df = 8 (P < 0.0001); I² = 78%				
Test for overall effect: Z = 1.63 (P = 0.10)				
**1.4.2 Ischemic stroke**				
Baratta 2020	-0.5226	0.9159	0.3%	0.59 [0.10, 3.57]
El Azeem 2013(a)	0.824	0.2362	3.3%	2.28 [1.43, 3.62]
Fracanzani 2016	1.4202	0.8758	0.3%	4.14 [0.74, 23.03]
Hamaguchi 2007(a)	1.4755	0.5821	0.7%	4.37 [1.40, 13.69]
Kwak 2018	0.7129	0.3487	1.7%	2.04 [1.03, 4.04]
Lee H 2021	0.0296	0.0359	14.2%	1.03 [0.96, 1.11]
Moshayedi 2014	0.5188	0.7073	0.5%	1.68 [0.42, 6.72]
Weinstein 2018	0.4947	0.3353	1.8%	1.64 [0.85, 3.16]
Xu 2021	0.1484	0.0412	13.9%	1.16 [1.07, 1.26]
**Subtotal (95% CI)**			**36.6%**	**1.35 [1.11, 1.63]**
Heterogeneity: Tau² = 0.03; Chi² = 27.76, df = 8 (P = 0.0005); I² = 71%				
Test for overall effect: Z = 3.02 (P = 0.002)				
**1.4.3 Hemorrhagic stroke**				
El Azeem 2013(b)	0.5801	0.2966	2.3%	1.79 [1.00, 3.19]
Hamaguchi 2007(b)	1.4586	1.4161	0.1%	4.30 [0.27, 69.00]
**Subtotal (95% CI)**			**2.4%**	**1.85 [1.05, 3.27]**
Heterogeneity: Tau² = 0.00; Chi² = 0.37, df = 1 (P = 0.54); I² = 0%				
Test for overall effect: Z = 2.13 (P = 0.03)				
**Total (95% CI)**			**100.0%**	**1.18 [1.08, 1.30]**
Heterogeneity: Tau² = 0.02; Chi² = 68.08, df = 19 (P < 0.00001); I² = 72%				
Test for overall effect: Z = 3.48 (P = 0.0005)				
Test for subgroup differences: Chi² = 4.94, df = 2 (P = 0.08), I² = 59.5%				

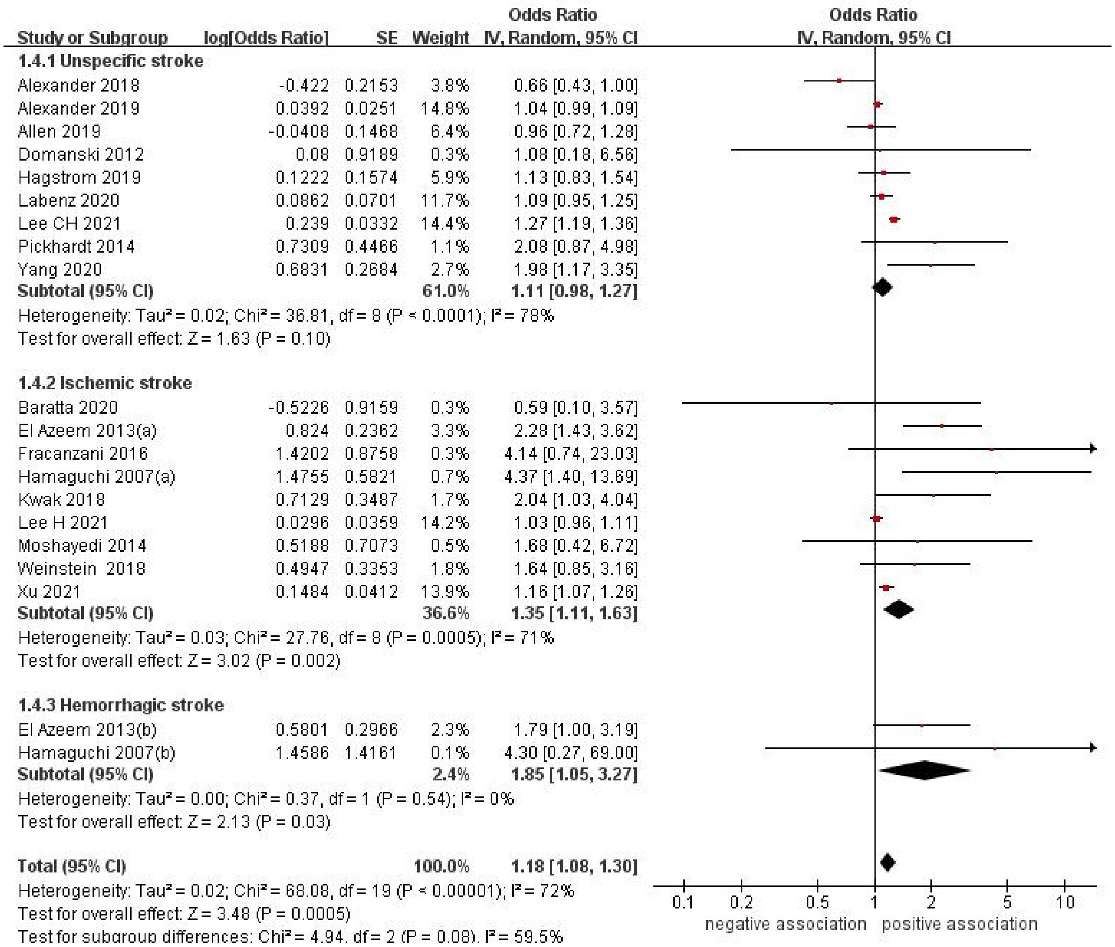

Figure S3. Forest plot of subgroup analysis stratified by type of stroke

Study or Subgroup	log[Odds Ratio]	SE	Weight	Odds Ratio IV, Random, 95% CI
**1.5.1 Imaging techniques**				
Baratta 2020	-0.5226	0.9159	0.3%	0.59 [0.10, 3.57]
El Azeem 2013(a)	0.824	0.2362	3.3%	2.28 [1.43, 3.62]
El Azeem 2013(b)	0.5801	0.2966	2.3%	1.79 [1.00, 3.19]
Fracanzani 2016	1.4202	0.8758	0.3%	4.14 [0.74, 23.03]
Hamaguchi 2007(a)	1.4755	0.5821	0.7%	4.37 [1.40, 13.69]
Hamaguchi 2007(b)	1.4586	1.4161	0.1%	4.30 [0.27, 69.00]
Kwak 2018	0.7129	0.3487	1.7%	2.04 [1.03, 4.04]
Moshayedi 2014	0.5188	0.7073	0.5%	1.68 [0.42, 6.72]
Pickhardt 2014	0.7309	0.4466	1.1%	2.08 [0.87, 4.98]
Weinstein 2018	0.4947	0.3353	1.8%	1.64 [0.85, 3.16]
Xu 2021	0.1484	0.0412	13.9%	1.16 [1.07, 1.26]
**Subtotal (95% CI)**			**25.9%**	**1.81 [1.31, 2.49]**
Heterogeneity: Tau² = 0.11; Chi² = 22.90, df = 10 (P = 0.01); I² = 56%				
Test for overall effect: Z = 3.64 (P = 0.0003)				
**1.5.2 Diagnostic codes**				
Alexander 2019	0.0392	0.0251	14.8%	1.04 [0.99, 1.09]
Allen 2019	-0.0408	0.1468	6.4%	0.96 [0.72, 1.28]
Labenz 2020	0.0862	0.0701	11.7%	1.09 [0.95, 1.25]
**Subtotal (95% CI)**			**32.8%**	**1.04 [1.00, 1.09]**
Heterogeneity: Tau² = 0.00; Chi² = 0.73, df = 2 (P = 0.69); I² = 0%				
Test for overall effect: Z = 1.82 (P = 0.07)				
**1.5.3 Fatty liver index**				
Alexander 2018	-0.422	0.2153	3.8%	0.66 [0.43, 1.00]
Lee CH 2021	0.239	0.0332	14.4%	1.27 [1.19, 1.36]
Lee H 2021	0.0296	0.0359	14.2%	1.03 [0.96, 1.11]
Yang 2020	0.6831	0.2684	2.7%	1.98 [1.17, 3.35]
**Subtotal (95% CI)**			**35.1%**	**1.12 [0.91, 1.38]**
Heterogeneity: Tau² = 0.03; Chi² = 29.26, df = 3 (P < 0.00001); I² = 90%				
Test for overall effect: Z = 1.06 (P = 0.29)				
**1.5.4 Liver biopsy**				
Domanski 2012	0.08	0.9189	0.3%	1.08 [0.18, 6.56]
Hagstrom 2019	0.1222	0.1574	5.9%	1.13 [0.83, 1.54]
**Subtotal (95% CI)**			**6.1%**	**1.13 [0.83, 1.53]**
Heterogeneity: Tau² = 0.00; Chi² = 0.00, df = 1 (P = 0.96); I² = 0%				
Test for overall effect: Z = 0.78 (P = 0.44)				
**Total (95% CI)**			**100.0%**	**1.18 [1.08, 1.30]**
Heterogeneity: Tau² = 0.02; Chi² = 68.08, df = 19 (P < 0.00001); I² = 72%				
Test for overall effect: Z = 3.48 (P = 0.0005)				
Test for subgroup differences: Chi² = 11.65, df = 3 (P = 0.009), I² = 74.2%				

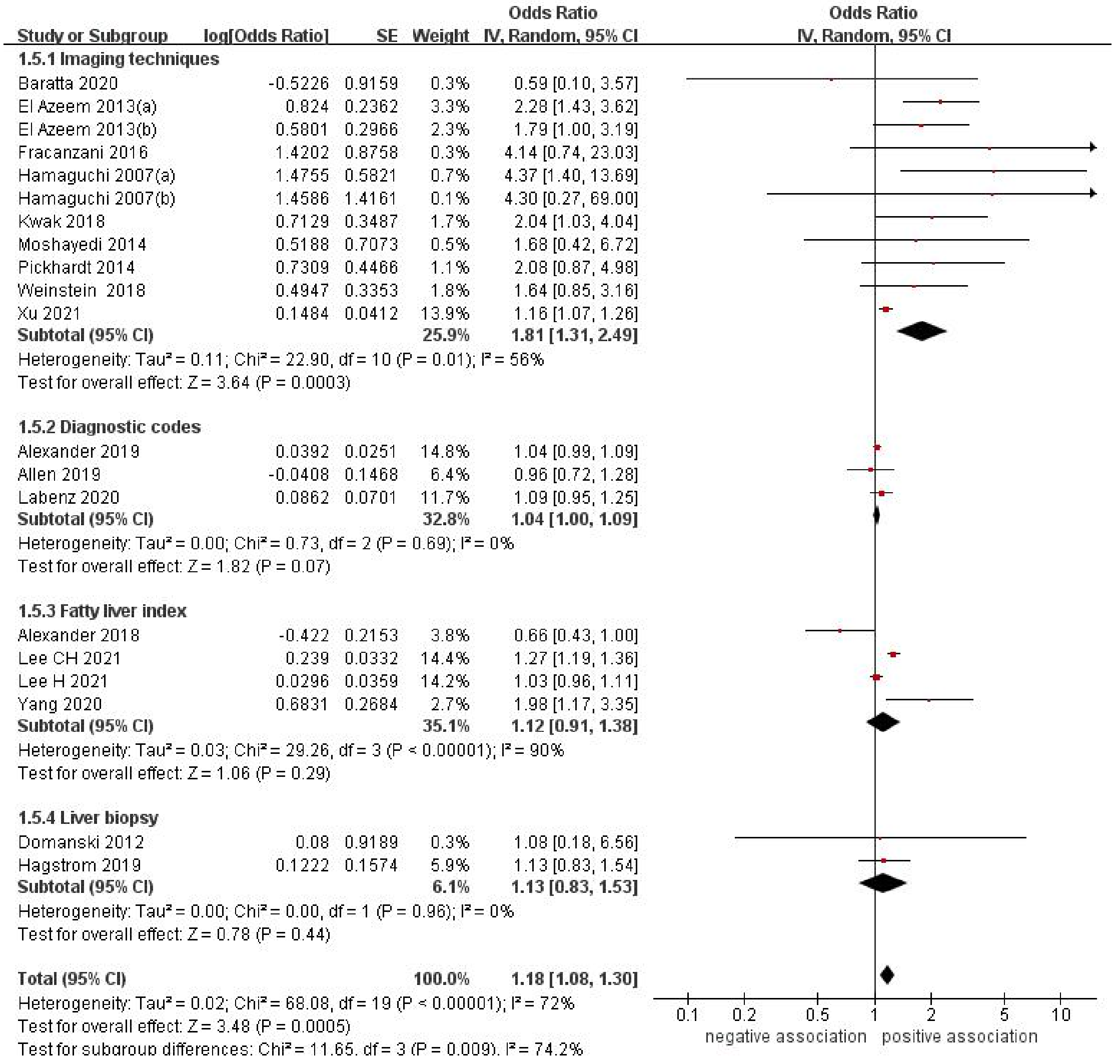

Figure S4. Forest plot of subgroup analysis stratified by diagnostic methods of NAFLD

Study or Subgroup	log[Odds Ratio]	SE	Weight	Odds Ratio IV, Random, 95% CI
**1.6.1 ≥65 years**				
Alexander 2018	-0.422	0.2153	3.9%	0.66 [0.43, 1.00]
Moshayedi 2014	0.5188	0.7073	0.5%	1.68 [0.42, 6.72]
Weinstein 2018	0.4947	0.3353	1.9%	1.64 [0.85, 3.16]
**Subtotal (95% CI)**			**6.2%**	**1.09 [0.52, 2.27]**
Heterogeneity: Tau² = 0.27; Chi² = 6.14, df = 2 (P = 0.05); I² = 67%				
Test for overall effect: Z = 0.24 (P = 0.81)				
**1.6.2 <65 years**				
Alexander 2019	0.0392	0.0251	14.8%	1.04 [0.99, 1.09]
Allen 2019	-0.0408	0.1468	6.4%	0.96 [0.72, 1.28]
Baratta 2020	-0.5226	0.9159	0.3%	0.59 [0.10, 3.57]
El Azeem 2013(a)	0.824	0.2362	3.3%	2.28 [1.43, 3.62]
El Azeem 2013(b)	0.5801	0.2966	2.3%	1.79 [1.00, 3.19]
Fracanzani 2016	1.4202	0.8758	0.3%	4.14 [0.74, 23.03]
Hagstrom 2019	0.1222	0.1574	5.9%	1.13 [0.83, 1.54]
Hamaguchi 2007(a)	1.4755	0.5821	0.7%	4.37 [1.40, 13.69]
Hamaguchi 2007(b)	1.4586	1.4161	0.1%	4.30 [0.27, 69.00]
Kwak 2018	0.7129	0.3487	1.7%	2.04 [1.03, 4.04]
Labenz 2020	0.0862	0.0701	11.7%	1.09 [0.95, 1.25]
Lee CH 2021	0.239	0.0332	14.3%	1.27 [1.19, 1.36]
Lee H 2021	0.0296	0.0359	14.2%	1.03 [0.96, 1.11]
Pickhardt 2014	0.7309	0.4466	1.1%	2.08 [0.87, 4.98]
Xu 2021	0.1484	0.0412	13.9%	1.16 [1.07, 1.26]
Yang 2020	0.6831	0.2684	2.7%	1.98 [1.17, 3.35]
**Subtotal (95% CI)**			**93.8%**	**1.20 [1.09, 1.32]**
Heterogeneity: Tau² = 0.01; Chi² = 60.36, df = 15 (P < 0.00001); I² = 75%				
Test for overall effect: Z = 3.72 (P = 0.0002)				
**Total (95% CI)**			**100.0%**	**1.18 [1.08, 1.30]**
Heterogeneity: Tau² = 0.02; Chi² = 68.08, df = 18 (P < 0.00001); I² = 74%				
Test for overall effect: Z = 3.47 (P = 0.0005)				
Test for subgroup differences: Chi² = 0.06, df = 1 (P = 0.81), I² = 0%				

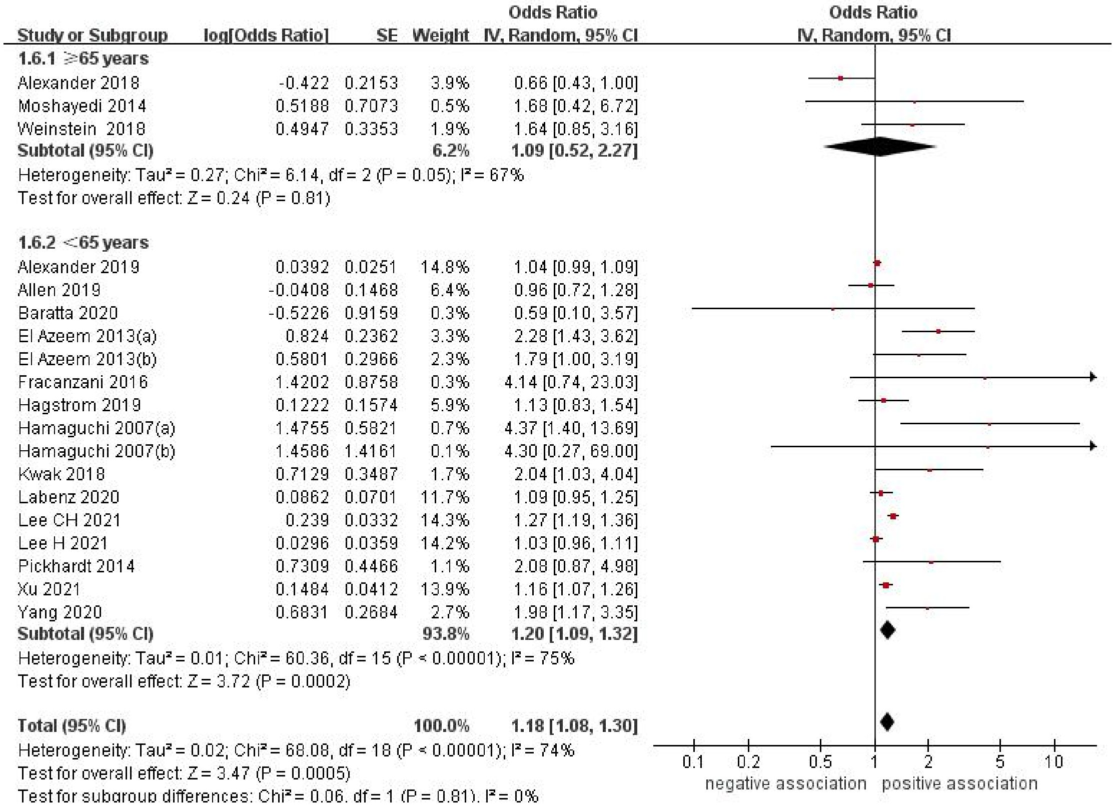

Figure S5. Forest plot of subgroup analysis stratified by mean age of study participants

Study or Subgroup	log[Odds Ratio]	SE	Weight	Odds Ratio IV, Random, 95% CI
**1.7.1 Male > Female**				
Alexander 2019	0.0392	0.0251	14.8%	1.04 [0.99, 1.09]
Baratta 2020	-0.5226	0.9159	0.3%	0.59 [0.10, 3.57]
Domanski 2012	0.08	0.9189	0.3%	1.08 [0.18, 6.56]
Fracanzani 2016	1.4202	0.8758	0.3%	4.14 [0.74, 23.03]
Hagstrom 2019	0.1222	0.1574	5.9%	1.13 [0.83, 1.54]
Hamaguchi 2007(a)	1.4755	0.5821	0.7%	4.37 [1.40, 13.69]
Hamaguchi 2007(b)	1.4586	1.4161	0.1%	4.30 [0.27, 69.00]
Kwak 2018	0.7129	0.3487	1.7%	2.04 [1.03, 4.04]
Labenz 2020	0.0862	0.0701	11.7%	1.09 [0.95, 1.25]
Lee CH 2021	0.239	0.0332	14.4%	1.27 [1.19, 1.36]
Moshayedi 2014	0.5188	0.7073	0.5%	1.68 [0.42, 6.72]
Xu 2021	0.1484	0.0412	13.9%	1.16 [1.07, 1.26]
**Subtotal (95% CI)**			**64.4%**	**1.17 [1.06, 1.30]**
Heterogeneity: Tau² = 0.01; Chi² = 36.14, df = 11 (P = 0.0002); I² = 70%				
Test for overall effect: Z = 2.97 (P = 0.003)				
**1.7.2 Male < Female**				
Alexander 2018	-0.422	0.2153	3.8%	0.66 [0.43, 1.00]
Allen 2019	-0.0408	0.1468	6.4%	0.96 [0.72, 1.28]
El Azeem 2013(a)	0.824	0.2362	3.3%	2.28 [1.43, 3.62]
El Azeem 2013(b)	0.5801	0.2966	2.3%	1.79 [1.00, 3.19]
Lee H 2021	0.0296	0.0359	14.2%	1.03 [0.96, 1.11]
Pickhardt 2014	0.7309	0.4466	1.1%	2.08 [0.87, 4.98]
Weinstein 2018	0.4947	0.3353	1.8%	1.64 [0.85, 3.16]
Yang 2020	0.6831	0.2684	2.7%	1.98 [1.17, 3.35]
**Subtotal (95% CI)**			**35.6%**	**1.32 [1.01, 1.74]**
Heterogeneity: Tau² = 0.10; Chi² = 29.05, df = 7 (P = 0.0001); I² = 76%				
Test for overall effect: Z = 2.01 (P = 0.04)				
**Total (95% CI)**			**100.0%**	**1.18 [1.08, 1.30]**
Heterogeneity: Tau² = 0.02; Chi² = 68.08, df = 19 (P < 0.00001); I² = 72%				
Test for overall effect: Z = 3.48 (P = 0.0005)				
Test for subgroup differences: Chi² = 0.65, df = 1 (P = 0.42), I² = 0%				

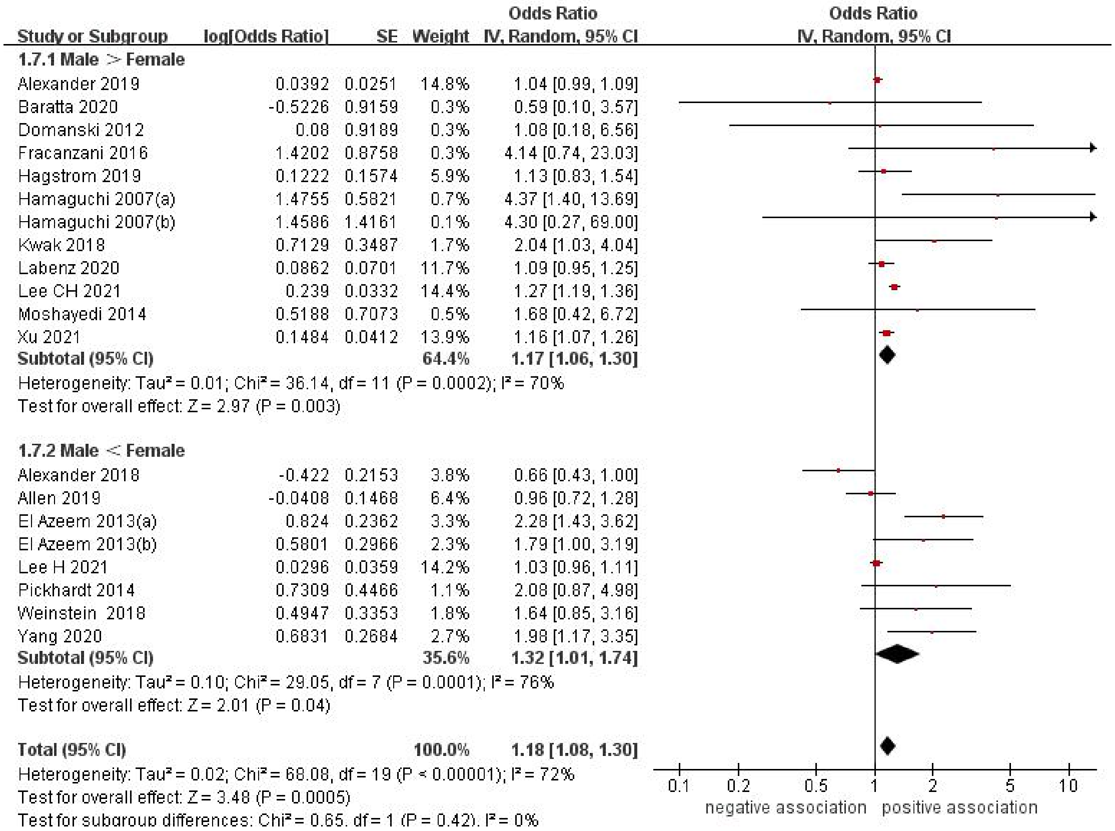

Figure S6. Forest plot of subgroup analysis stratified by number of study participants sex

Study or Subgroup	log[]	SE	Weight	IV, Random, 95% CI
**1.8.1 High quality**				
Alexander 2018	-0.422	0.2153	3.8%	0.66 [0.43, 1.00]
Alexander 2019	0.0392	0.0251	14.8%	1.04 [0.99, 1.09]
Allen 2019	-0.0408	0.1468	6.4%	0.96 [0.72, 1.28]
Fracanzani 2016	1.4202	0.8758	0.3%	4.14 [0.74, 23.03]
Hagstrom 2019	0.1222	0.1574	5.9%	1.13 [0.83, 1.54]
Kwak 2018	0.7129	0.3487	1.7%	2.04 [1.03, 4.04]
Labenz 2020	0.0862	0.0701	11.7%	1.09 [0.95, 1.25]
Lee CH 2021	0.239	0.0332	14.4%	1.27 [1.19, 1.36]
Lee H 2021	0.0296	0.0359	14.2%	1.03 [0.96, 1.11]
Moshayedi 2014	0.5188	0.7073	0.5%	1.68 [0.42, 6.72]
Weinstein 2018	0.4947	0.3353	1.8%	1.64 [0.85, 3.16]
Xu 2021	0.1484	0.0412	13.9%	1.16 [1.07, 1.26]
Yang 2020	0.6831	0.2684	2.7%	1.98 [1.17, 3.35]
**Subtotal (95% CI)**			**92.0%**	**1.12 [1.03, 1.23]**
Heterogeneity: Tau² = 0.01; Chi² = 47.29, df = 12 (P < 0.00001); I² = 75%				
Test for overall effect: Z = 2.58 (P = 0.010)				
**1.8.2 Moderate quality**				
Baratta 2020	-0.5226	0.9159	0.3%	0.59 [0.10, 3.57]
Domanski 2012	0.08	0.9189	0.3%	1.08 [0.18, 6.56]
El Azeem 2013(a)	0.824	0.2362	3.3%	2.28 [1.43, 3.62]
El Azeem 2013(b)	0.5801	0.2966	2.3%	1.79 [1.00, 3.19]
Hamaguchi 2007(a)	1.4755	0.5821	0.7%	4.37 [1.40, 13.69]
Hamaguchi 2007(b)	1.4586	1.4161	0.1%	4.30 [0.27, 69.00]
Pickhardt 2014	0.7309	0.4466	1.1%	2.08 [0.87, 4.98]
**Subtotal (95% CI)**			**8.0%**	**2.09 [1.53, 2.85]**
Heterogeneity: Tau² = 0.00; Chi² = 4.69, df = 6 (P = 0.58); I² = 0%				
Test for overall effect: Z = 4.67 (P < 0.00001)				
**Total (95% CI)**			**100.0%**	**1.18 [1.08, 1.30]**
Heterogeneity: Tau² = 0.02; Chi² = 68.08, df = 19 (P < 0.00001); I² = 72%				
Test for overall effect: Z = 3.48 (P = 0.0005)				
Test for subgroup differences: Chi² = 14.31, df = 1 (P = 0.0002), I² = 93.0%				

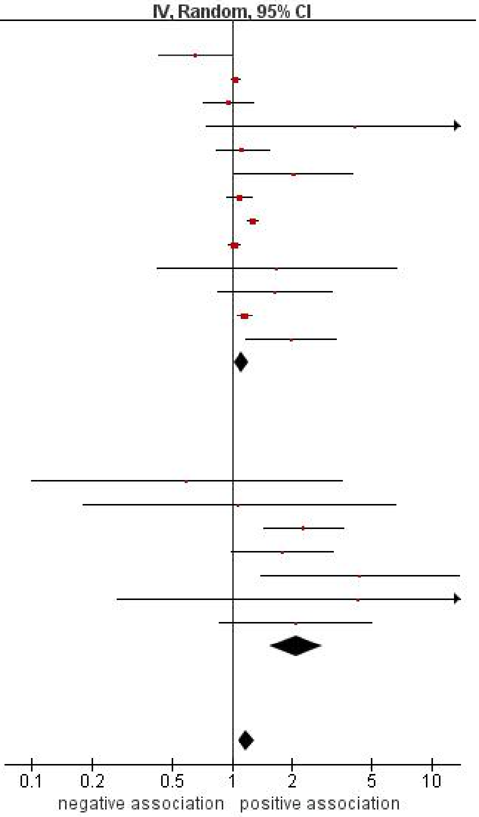

Figure S7. Forest plot of subgroup analysis stratified by study quality

Study or Subgroup	log[Odds Ratio]	SE	Weight	Odds Ratio IV, Random, 95% CI
**1.9.1 Adjusted OR**				
Alexander 2018	-0.422	0.2153	3.5%	0.66 [0.43, 1.00]
Alexander 2019	0.0392	0.0251	17.2%	1.04 [0.99, 1.09]
Allen 2019	-0.0408	0.1468	6.2%	0.96 [0.72, 1.28]
Fracanzani 2016	1.4202	0.8758	0.3%	4.14 [0.74, 23.03]
Hagstrom 2019	0.1222	0.1574	5.6%	1.13 [0.83, 1.54]
Kwak 2018	0.7129	0.3487	1.5%	2.04 [1.03, 4.04]
Labenz 2020	0.0862	0.0701	12.6%	1.09 [0.95, 1.25]
Lee CH 2021	0.239	0.0332	16.5%	1.27 [1.19, 1.36]
Lee H 2021	0.0296	0.0359	16.3%	1.03 [0.96, 1.11]
Moshayedi 2014	0.5188	0.7073	0.4%	1.68 [0.42, 6.72]
Weinstein 2018	0.4947	0.3353	1.6%	1.64 [0.85, 3.16]
Xu 2021	0.1484	0.0412	15.8%	1.16 [1.07, 1.26]
Yang 2020	0.6831	0.2684	2.4%	1.98 [1.17, 3.35]
**Subtotal (95% CI)**			**100.0%**	**1.12 [1.03, 1.23]**
Heterogeneity: Tau² = 0.01; Chi² = 47.29, df = 12 (P < 0.00001); I² = 75%				
Test for overall effect: Z = 2.58 (P = 0.010)				
**1.9.2 Unadjusted OR**				
Allen 2019	0.2677	0.0792	17.4%	1.31 [1.12, 1.53]
Baratta 2020	-0.5226	0.9159	1.1%	0.59 [0.10, 3.57]
Domanski 2012	0.08	0.9189	1.1%	1.08 [0.18, 6.56]
El Azeem 2013(a)	0.824	0.2362	9.1%	2.28 [1.43, 3.62]
El Azeem 2013(b)	0.5801	0.2966	6.9%	1.79 [1.00, 3.19]
Hagstrom 2019	0.0052	0.1561	13.0%	1.01 [0.74, 1.36]
Hamaguchi 2007(a)	1.4755	0.5821	2.4%	4.37 [1.40, 13.69]
Hamaguchi 2007(b)	1.4586	1.4161	0.5%	4.30 [0.27, 69.00]
Lee CH 2021	0.5766	0.0295	19.3%	1.78 [1.68, 1.89]
Moshayedi 2014	0.9308	0.2982	6.9%	2.54 [1.41, 4.55]
Pickhardt 2014	0.7309	0.4466	3.8%	2.08 [0.87, 4.98]
Weinstein 2018	0.0899	0.2686	7.9%	1.09 [0.65, 1.85]
Yang 2020	0.6523	0.2029	10.6%	1.92 [1.29, 2.86]
**Subtotal (95% CI)**			**100.0%**	**1.62 [1.34, 1.96]**
Heterogeneity: Tau² = 0.05; Chi² = 36.03, df = 12 (P = 0.0003); I² = 67%				
Test for overall effect: Z = 4.94 (P < 0.00001)				

Test for subgroup differences: Chi² = 11.57, df = 1 (P = 0.0007), I² = 91.4%

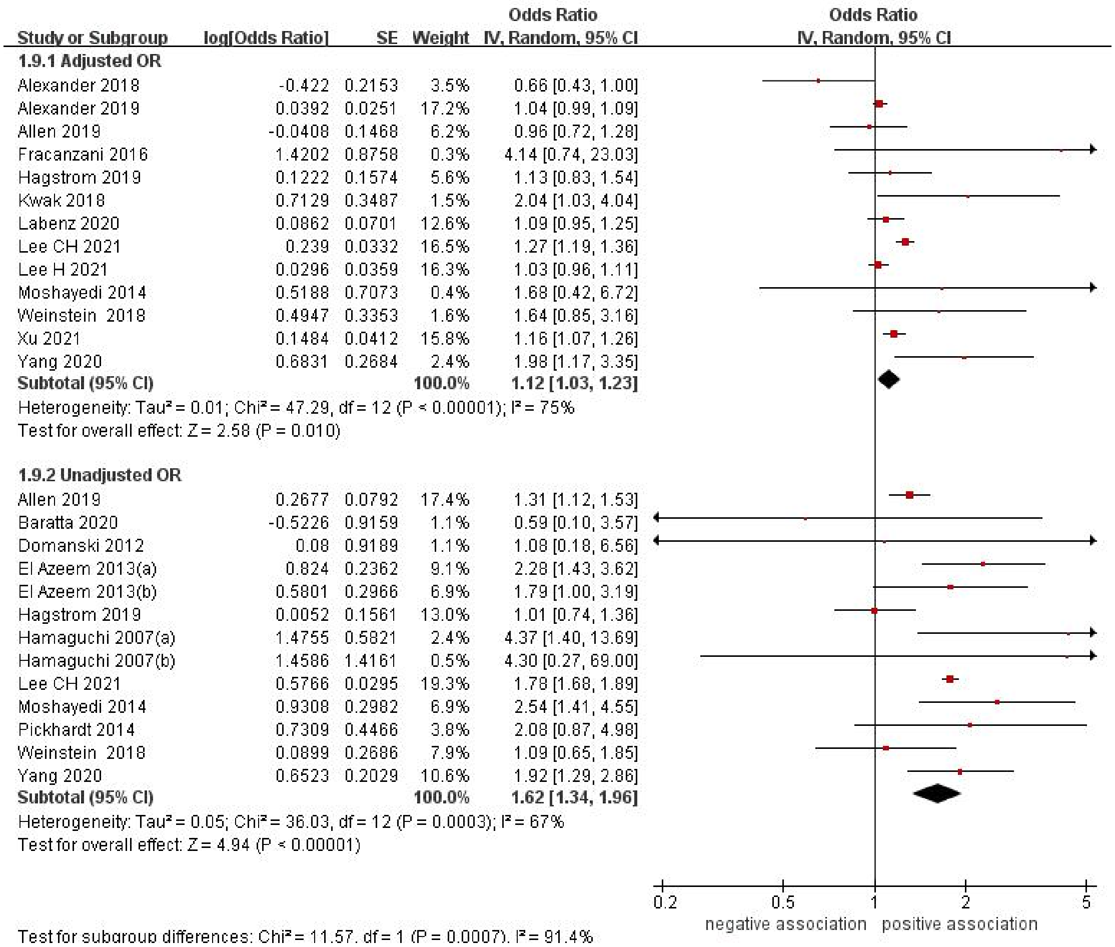

Figure S8. Forest plot of subgroup analysis stratified by adjustment for confounders

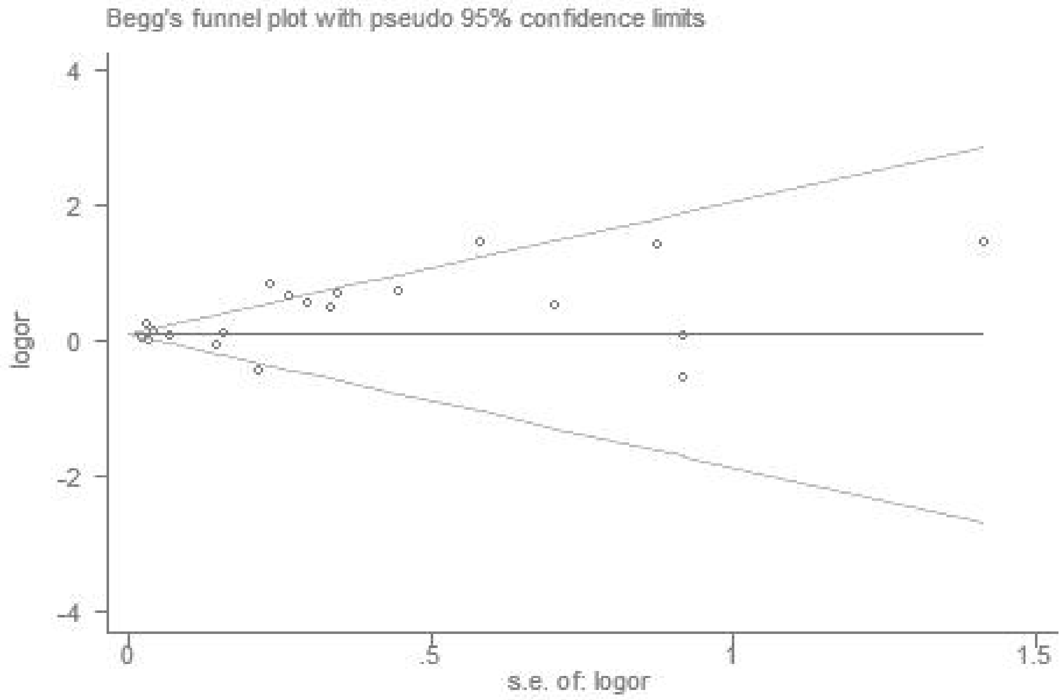

Figure S9. Begg's funnel plot for association between NAFLD and risk of stroke
